# Sex bias consideration in healthcare machine-learning research: a systematic review in rheumatoid arthritis

**DOI:** 10.1136/bmjopen-2024-086117

**Published:** 2025-03-13

**Authors:** Anahita Talwar, Shruti Turner, Claudia Maw, Georgina Quayle, Thomas N Watt, Sunir Gohil, Emma Duckworth, Coziana Ciurtin

**Affiliations:** 1 Haleon plc, Weybridge, UK; 2 Department of Rheumatology, University College London, London, UK

**Keywords:** Machine Learning, STATISTICS & RESEARCH METHODS, Health Equity, RHEUMATOLOGY, MEDICAL ETHICS

## Abstract

**Abstract:**

**Objective:**

To assess the acknowledgement and mitigation of sex bias within studies using supervised machine learning (ML) for improving clinical outcomes in rheumatoid arthritis (RA).

**Design:**

A systematic review of original studies published in English between 2018 and November 2023.

**Data sources:**

PUBMED and EMBASE databases.

**Study selection:**

Studies were selected based on their use of supervised ML in RA and their publication within the specified date range.

**Data extraction and synthesis:**

Papers were scored on whether they reported, attempted to mitigate or successfully mitigated various types of bias: training data bias, test data bias, input variable bias, output variable bias and analysis bias. The quality of ML research in all papers was also assessed.

**Results:**

Out of 52 papers included in the review, 51 had a female skew in their study participants. However, 42 papers did not acknowledge any potential sex bias. Only three papers assessed bias in model performance by sex disaggregating their results. Potential sex bias in input variables was acknowledged in one paper, while six papers commented on sex bias in their output variables, predominantly disease activity scores. No paper attempted to mitigate any type of sex bias.

**Conclusions:**

The findings demonstrate the need for increased promotion of inclusive and equitable ML practices in healthcare to address unchecked sex bias in ML algorithms.

**PROSPERO registration number:**

CRD42023431754.

**STRENGTHS AND LIMITATIONS OF THIS STUDY**

- Comprehensive search strategy employed for systematic review.
- Inclusion of diverse study populations and methodologies enhances generalisability.
- Rigorous application of inclusion/exclusion criteria across the dataset.
- Transparency in assessing and acknowledging potential biases.
- Limitations include potential selection bias due to publication bias and language restriction to English studies, and challenges in delineating sex and gender features across included papers.

## Introduction

 Machine-learning (ML) algorithms—computational models and techniques that use patterns learnt from data to make useful predictions—hold immense potential for advancing precision medicine in healthcare. The applications of ML remain wide, including disease diagnosis, pattern or image recognition and classification, drug development, and prognostic prediction tools for clinical use.^1^ The implementation of ML models into real-world settings raises ethical concerns that necessitate careful consideration during model development to produce fair algorithms.^2^,^4^ ML algorithms learn from existing data, and if the data contain biases or imbalances, they can result in inequalities in the model’s predictions, ultimately affecting the model’s clinical utility and relevance for the wider population. Thus, ensuring the equity and fairness of developed algorithms when implemented into real-world settings is crucial and commands the consideration of often overlooked individual differences in healthcare, including sex and gender disparities.

### Sex bias in ML research in healthcare

It is now widely acknowledged that sex and gender are distinct but interconnected concepts, with sex referring to the biological characteristics, primarily chromosomes, reproductive organs and hormones, and gender referring to broader social, cultural and psychological aspects an individual identifies with. There has been an increasing emphasis on sex and gender as variables in analysis in healthcare research.^5^,^8^ This is in part driven by an evolving recognition of the substantial influence that sex and gender differences exert on symptomatology, disease phenotypes, treatment responses and experience of care.^9^,^12^ Additionally, it stems from an increased awareness of the historical underrepresentation of women in clinical and preclinical trials, as well as in research professions,^13 14^ and the consequential adverse implications for women in clinical practice.^15 16^ When parameters related to, derived from or significantly impacted by an individual’s sex are successfully accounted for in model development, ML algorithms can contribute to improved equity in the quality of care for both sexes. This is exemplified by AwareDx, which uses a random forest algorithm to predict sex differences in drug response to help physicians provide tailored drug dosing and minimise adverse events.^17^ When bias in such models is disregarded or unsuccessfully mitigated and used to guide clinicians’ decisions in practice, it can result in inequitable care and treatment for patients of either sex and ultimately unfair outcomes.

A common source of bias arises when certain groups are under-represented in training and test data, contributing to biased model predictions.^18 19^ While the impact of this under-representation is complex and context-dependent, influenced also by group differences and model training methods, it may result in poorer performance for these groups.^20^ Additionally, biases can originate from variables used as predictors in the model, such as healthcare measures that are more representative of symptoms experienced by one sex. When biased models are applied in conjunction with clinical practice, they can exacerbate disparities in diagnosis, treatment decisions and access to care, perpetuating inequities in healthcare. To address this issue proactively, it is essential to critically evaluate and refine ML algorithms, ensuring they are sensitive to and account for sex, as well as gender differences. By mitigating bias in algorithmic decision-making processes, healthcare systems can strive for more equitable and inclusive care delivery, ultimately improving health outcomes for all individuals.

Rheumatoid arthritis (RA) is a strong candidate for exploring sex bias in ML due to its relatively high prevalence (approximately 1%) in the general population,^21^ female predominance irrespective of age at onset, as well as relative abundance of studies including ML algorithms in the literature.

### Using RA to explore gender bias in ML

RA is a chronic autoimmune disease, with an estimated 17.8 million individuals living with it worldwide in 2020.^22^ It is generally characterised by inflammation of the synovial joints, leading to pain, stiffness and swelling, which severely impact mobility and quality of life. However, RA is a heterogeneous condition with individuals exhibiting diverse immunomolecular profiles and clinical phenotypes,^23 24^ varying responses to established and emerging treatments^25^,^27^ and complexities in comorbidity management, despite clear benefits of prompt diagnosis and pharmacological intervention.^28^,^32^ ML offers a promising contribution to optimal RA management by identifying complex patterns in patient data (eg, reported symptoms, genetics, blood and imaging biomarkers), and improving predictions related to patient outcomes.^33^,^36^ Finally, sex differences have been reported in RA, such as a threefold increased prevalence in women versus men overall, poorer symptom scores, greater impact on quality of life and increased prevalence of irreversible joint damage in women compared with men,^37 38^ as well as reduced treatment efficacy and adherence in women.^39^,^42^ There are several potential and interdependent explanations for the observed sex differences in RA presentation, including the influence of sex hormones, discrepancies in the processing and reporting of pain, sex-specific applicability of disease activity measurements and bias in healthcare delivery.^38 43^ These highlight the need to consider such factors when devising personalised management plans and treatment recommendations, and particularly when incorporating ML into practice due to its ability to exacerbate such differences.

### Investigating the extent to which sex bias is considered in ML research in RA

The objective of this systematic review is to investigate the extent to which sex bias is considered and mitigated in ML research related to RA. We focus on five distinct types of bias, specifically:

1. Training data bias: This bias stems from sex skew in the training datasets, leading to models that may perform differently for men and women.
2. Test data bias: This bias originates from sex skew in the test dataset. It affects the evaluation of the model’s performance, potentially leading to misleading conclusions about its effectiveness across sexes.
3. Input variable bias: This bias arises from input variables that may have different distributions or levels of informativeness between sexes, conditioned on disease status. It may be compounded by label/measurement bias, which occurs when there are systematic errors in the way data are labelled or measured.
4. Output variable bias: This bias is associated with output variables that may have different distributions or levels of informativeness between sexes, conditioned on disease status. It may be compounded by label/measurement bias.
5. Analysis bias: This bias results from the omission of sex-disaggregated analyses, potentially masking important variations and leading to biased conclusions.

By examining these aspects, we seek to shed light on which of these potential biases are considered during ML model development in the domain of RA, how often they occur and ultimately strive to improve responsible artificial intelligence (AI) and transparency in mitigating bias and ensuring equitable outcomes in healthcare.

## Methods

The reporting of this systematic review was guided by the standards of the Preferred Reporting Items for Systematic Reviews and Meta-Analyses (PRISMA) Statement. The protocol for this systematic review was preregistered on PROSPERO with ID CRD42023431754.

### Search strategy

The search strategy was defined to identify research studies using ML in the domain of RA from two electronic databases, PubMed and EMBASE. Search terms related to ML and RA were combined into the following search strategy: (machine learning OR ML OR artificial intelligence OR AI OR deep learning) AND (rheumatoid arthritis OR RA OR rheumato* arthrit* OR rheumato* joint inflamm*). Searches were restricted to articles in English published between January 2018 and May 2023 (when the searches were run) to capture the most recent 5 years of research in the rapidly developing field of ML research. References from relevant review papers were explored, and the search was rerun in November 2023 before finalising the review to identify additional papers.

### Study selection

The inclusion and exclusion criteria (table 1) were consistently and iteratively applied to filter search results. The review focused on supervised ML papers and biological sex (rather than sex and gender) reporting to support consistency in the application of the bias-checking matrix. Further, we included papers which applied ML in the context of prediction (due to their more immediate applicability in clinical practice) rather than on inferential statistical analysis (conclusions around associations or relationships between variables) and used this rule for papers implementing methods that can be used for both (eg, linear and logistic regression).

**Table 1:** Inclusion and exclusion criteria for paper screening

	Inclusion	Exclusion
Type	T_IN_1: Original research studies.	T_EX_1: Review papers, meta-analyses, preprints, conference abstracts, inaccessible.
Scope	S_IN_1: Studies developing supervised machine learning models for the prediction of clinically relevant outcomes in rheumatoid arthritis.	S_EX_1: Studies applying existing machine learning models without developing new models in the domain of rheumatoid arthritis.
		S_EX_2: Studies lacking substantial focus on supervised machine learning methods including those utilising text mining, unsupervised learning or those reporting competition results.
		S_EX_3: Studies that integrate machine learning methods in rheumatoid arthritis without a primary focus on predicting clinically relevant outcomes.
Participants	P_IN_1: Studies including human participants with a confirmed diagnosis of any form of rheumatoid arthritis, diagnosed using recognised diagnostic criteria.	P_EX_1: Studies not involving humans (eg, animals, in vitro/ex vivo models, pharmacological agents).
		P_EX_2: Studies where the primary focus is not on rheumatoid arthritis (eg, only healthy controls, involves other autoimmune or musculoskeletal condition outcomes, focus on comorbidities).
Context		C_EX_1: Studies that do not mention sex.
		C_EX_2: Studies that include exclusively male or female participants.

The first letter of the code indicates the category of the criteria with T: type, S: scope, P: participants, C: context. The codes ‘IN’ and ‘EX’ represent inclusion and exclusion criteria, respectively, and the numbers following IN or EX (eg, 1, 2, 3) uniquely identify each specific criterion within its category.

**Figure 1:**
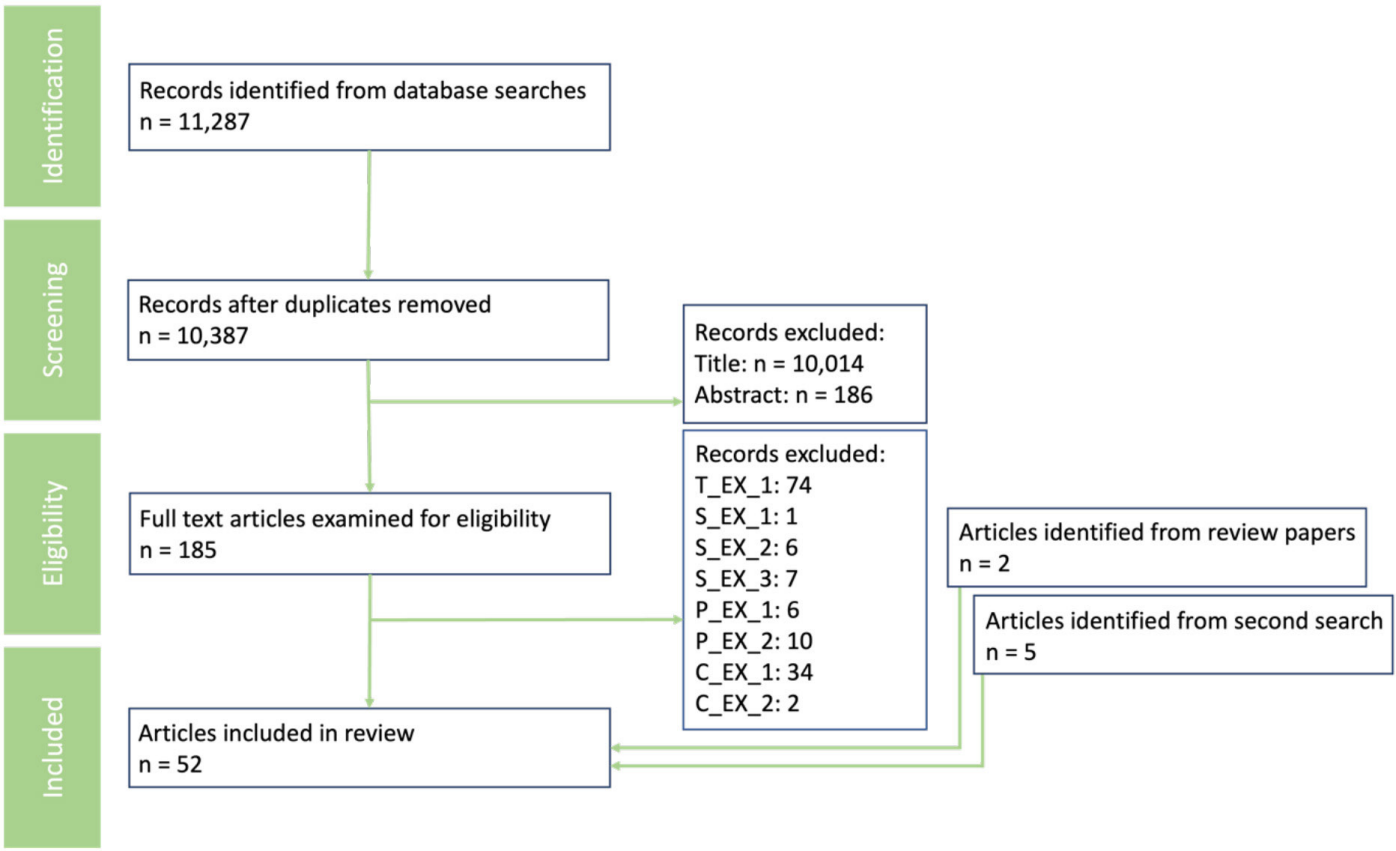
PRISMA flow diagram of study selection and screening process. See table 1 for the exclusion code definitions. PRISMA, Preferred Reporting Items for Systematic Review and Meta-Analyses.

Search results were exported to Mendeley and duplicate entries were removed. The subsequent stages of the review process were conducted by different authors, identified here by their initials. ST and CM conducted title and abstract screening to select relevant studies for further examination. AT and GQ independently screened the full-text version of the remaining papers for inclusion based on the eligibility criteria. Any disagreements between reviewers were resolved through discussion with SG. The PRISMA flow diagram (figure 1) provides a visual representation of the study selection process. 45 papers were included from the initial search and screening, with a further two identified from review papers, and a further five identified when the searches were rerun.

AT, ST, CM and GQ completed the data extraction and quality assessment from each of the full-text papers with AT checking scores to ensure consistent interpretation of the criteria. Any disagreements in scores were resolved through discussion with SG. All scores were recorded in an Excel spreadsheet (online supplemental table S1).

### Data extraction

For each of the full-text papers, the study objectives, types of ML method used and sex split of participants with RA were noted. Each paper was scored on whether the authors reported, attempted to mitigate or successfully mitigated the following types of bias: training data bias, test data bias, input variable bias, output variable bias, analysis bias, using a specifically designed scoring matrix (table 2). Our bias matrix was adapted from a framework focused on sex and gender bias in ML healthcare^4^ and tailored to be relevant and implementable in our systematic review context, and intuitive for readers. Coherent with our criteria, included papers were required to provide participants’ sex. Thus, for training and test data bias, we expected authors to comment on the impact of sex imbalance on model results to score 1. However, for the input and output variable bias and analysis bias, simply reporting how variables or analysis differed by sex was sufficient to score 1.

**Table 2:** Sex bias scoring matrix

Bias	Definition
Training data	This bias stems from sex skew in the training dataset, leading to models that may perform differently for men and women.
Test data	This bias originates from sex skew in the test dataset. It affects the evaluation of the model’s performance, potentially leading to misleading conclusions about its effectiveness across sexes.
Input variable	This bias arises from input variables that may have different distributions or levels of informativeness between sexes, conditioned on disease status. It may be compounded by label/measurement bias, which occurs when there are systematic errors in the way data is labelled or measured.
Output variable	This bias is associated with output variables that may have different distributions or levels of informativeness between sexes, conditioned on disease status. It may be compounded by label/measurement bias.
Analysis	This bias results from the omission of sex-disaggregated analyses, potentially masking important variations and leading to biased conclusions.
Score	Definition
N/A	Not applicable
0	Not mentioned
1	Reported but not mitigated, unsuccessfully mitigated or result of mitigation unclear
2	Successfully mitigated

In reviewing the literature, sex and gender terms are rarely distinguished or clarified. Thus, in this paper, it is assumed that the sex characteristic reflects the biological sex or that all participants are cis-gender due to the lack of clarity in all papers included in the final analysis.

### Quality assessment

The quality assessment matrix (table 3) was adapted and simplified from a previously published framework.^44^ Papers were scored on their implementation of the following methodologies: preprocessing decisions, validation data source, sample size, evaluation, hyperparameters and reproducibility.

**Table 3:** Quality assessment matrix

Category	Score	Definition
Preprocessing decisions	0	Not reported
	1	Partially reported
	2	Fully reported and replicable
Validation data source	0	Not done or used training data
	1	Used unseen sample from same dataset
	2	Used new sample
Sample size	0	No calculation or mention of sample size
	1	‘Big data’ hinted at, for example, commented on size of sample
	2	Sample size calculation done
Evaluation	0	Not done or accuracy metric only
	1	Accuracy metric plus error metrics of the accuracy (eg, CIs, or SE) or calibration
	2	All of accuracy, error metrics of the accuracy and calibration
Hyperparameters	0	No information
	1	Performed manually, random search or only report final values
	2	Optimised analytically or grid search
Reproducibility	0	None of data/code availability
	1	Some of data/code availability (statement or named dataset)
	2	All of data/code availability

### Data synthesis

The objectives of each study were identified and used to classify papers accordingly. The ML algorithms used for clinical prediction in each paper were also extracted and non-exclusively classified into broad categories. Summary statistics, including mean and SD of the percentage of female participants, were calculated across 51 of the 52 reviewed papers (including 1 paper that additionally incorporated participants with undifferentiated arthritis with the primary focus of predicting their transition to RA,^45^ and another that did not separate the sex split between the RA population and the healthy controls.^46^ We excluded one paper from this analysis as it only reported the training dataset sex split.^47^

### Data analysis

The Pearson’s correlation coefficient and associated p value between the quality scores and the consideration of sex bias scores were also calculated.

## Results

The screening process identified 52 papers (online supplemental table S1) fulfilling the inclusion/exclusion criteria, which were further subjected to qualitative synthesis and analysis.

### Characteristics of included papers

Data synthesis involved classifying the study objectives of papers. The resulting categories and their associated frequencies were: predict treatment response (23), score disease activity (11), improve diagnostic accuracy (10), assess joint damage (5) and identify patient subgroups (3).

**Figure 2:**
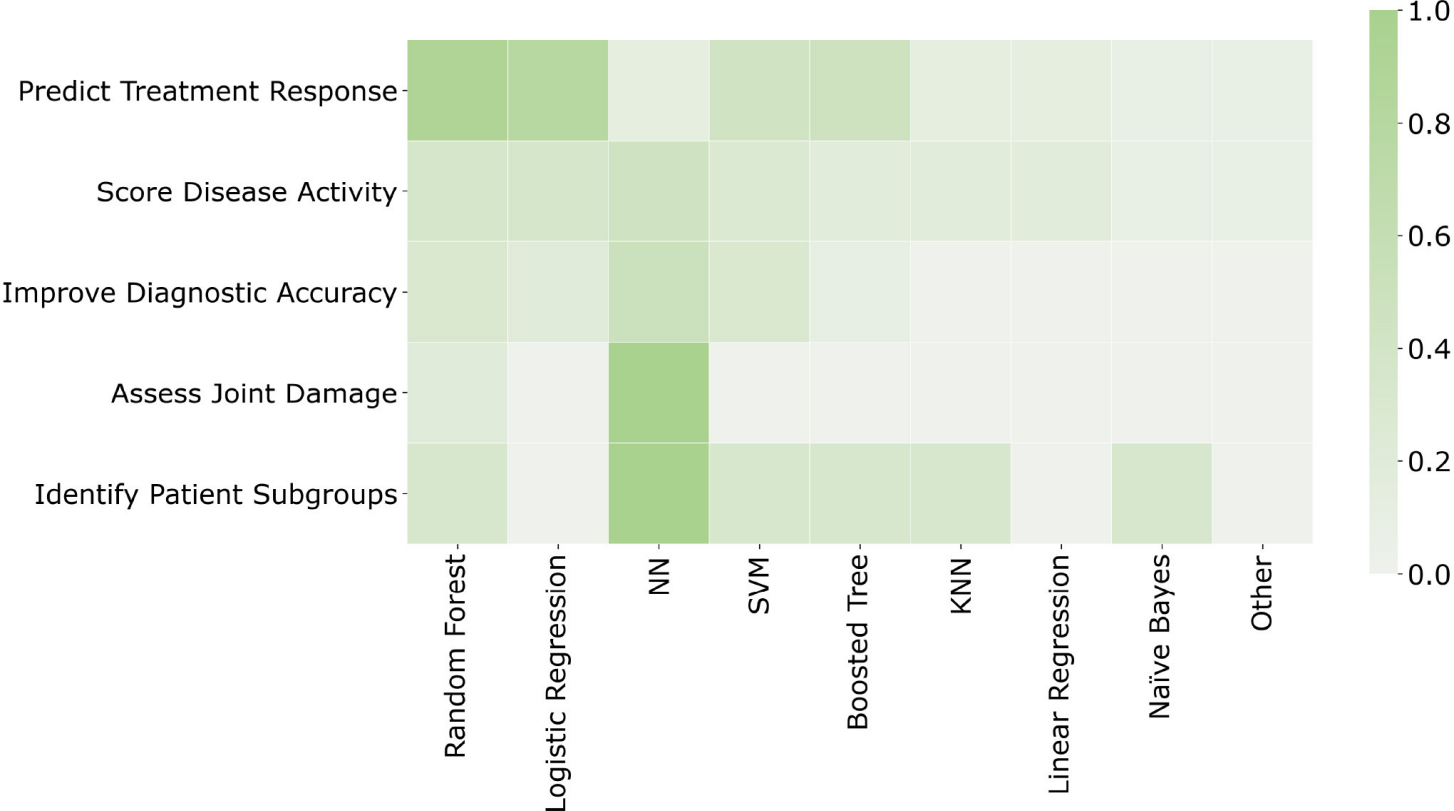
Proportion of papers with each study objective that used each type of ML model. KNN, K-nearest neighbours; ML, machine learning; NN, neural network; SVM, support vector machine.

The ML algorithms employed in each paper were non-exclusively grouped into broad categories. The resulting categories and their associated frequencies were random forest (30), logistic regression (24), neural networks (21), support vector machine (17), boosted tree (15), K-nearest neighbours (6), linear regression (5), naïve Bayes (4), other (3, including Gaussian process, pathway supported models and hidden Markov models). Numerous papers implemented and compared the performance of multiple algorithms, with some techniques more popular for certain study objectives such as neural networks for scoring disease activity and assessing joint damage (figure 2). In contrast, papers focused on improving diagnostic accuracy or identifying patient subgroups did not favour specific algorithms.

### Participant sex reporting and participant split

During full-text screening, 40 papers (6 from the repeated search) were excluded for not reporting the sex split of any RA participants (C_EX_1 in table 1). A further two papers were excluded for including only female participants (C_EX_2 in table 1). Of the included papers, the mean (SD) percentage of female participants was 74% (12%), consistent with the estimated global prevalence of RA at 70% female.^22^ All papers had a female recruitment bias in their participants, with the exception of 1 paper which only included 17% females (n=46) in the RA group.^48^

### Sex bias reporting and mitigation

After applying the bias checking matrix, 42 papers scored 0 across all sex bias categories. The remaining 10 papers each scored a total of 1 across all categories. Details on scores for specific types of bias are given below.

#### Training data bias & test data bias

No papers included in the final review acknowledged that sex bias, in either the training or test data, may have influenced the sex bias of their models’ predictions.

#### Input variable bias

Saleh *et al* statistically tested for sex differences in their model’s input variables, three bone biomarkers (osteopontin, stromelysin-1 (MMP3) and vascular endothelial growth factor-A) and reported no difference.^49^

#### Output variable bias

Potential sex bias in output variables was acknowledged by six papers, five with the objective of evaluating treatment response and one aimed at improving disease activity scoring. Sex-specific differences in baseline measures of erythrocyte sedimentation rate (ESR), often used as part of Disease Activity Scores (DAS), were mentioned by three papers in their discussion section. Myasoedova *et al* and Prasad *et al* suggest that women are less likely to achieve remission compared with men when using this measure,^50 51^ and Duong *et al* suggest that lack of age and sex-specific cut-offs for ESR may bias results.^52^

Statistical tests for sex differences in output variables were implemented in three papers. Curtis *et al* reported that sex differed meaningfully between participants who achieved low disease activity (defined as Clinical Disease Activity Index—CDAI<10) and those who did not.^53^ Tao *et al*, however, found no difference between responders and non-responders to adalimumab or etanercept treatment at baseline for clinical parameters (including sex) in either of their two cohorts of data,^54^ and Chen *et al* found no difference in response to leflunomide by sex.^55^

#### Analysis bias

Bias in model performance by sex disaggregation of results was assessed in three papers through different approaches. Bai *et al* presented the percentage of male participants for each type of outcome: True negative (57.9%, n=66), true positive (81.8%, n=72), false positive (75.0%, n=9) and false negative (66.7%, n=6), in a study aimed at improving diagnostic accuracy.^48^ The researchers used a dataset with 83.2% (n=225) males in the RA group and 59.4% (n=242) males in the control group, which likely explains the higher representation of males in the positive outcomes (both true and false) compared with the negative ones. However, this information does not actually tell us whether the model is equally accurate for diagnosing males and females overall, so the implications of these representation skews are unclear. Morales-Ivorra *et al* presented the AUROC (area under the receiver operating characteristic) results for the detection of active synovitis in different age and sex groups.^56^ Males (n=29) had an AUROC of 0.83 (95% CI 0.67 to 0.99; p<0.01), while females (n=117) had an AUROC of 0.77 (95% CI 0.68 to 0.85; p<0.01). Thus, despite having a strong female skew in the dataset, the authors described model performance as ‘similar’ across sexes without assessing this statistically. Finally, Kalweit *et al* statistically tested sex differences in performance of classification and regression algorithms for predicting DAS.^57^ While classification was more accurate for males, and regression more accurate for women, these differences were not statistically significant. However, it demonstrates an interesting point, that even when trained on the same data, different models exhibit varying accuracy across sex-based subgroups. This could be due to the different sensitivities of the models to particular data characteristics or specific training procedures, for example.

### Quality assessment

We conducted a quality assessment to evaluate the robustness of the data and the overall rigour of the research. A score of 0, 1 or 2 was given to each paper for each of seven quality metrics with equal weighting, thus giving a total quality score out of 12. Papers scoring 0–3 were deemed low quality, 4–8 medium and 9–12 considered high quality. Of the 52 papers, only 1 paper, Plant and Barton, was scored as high quality,^58^ 44 were determined to be medium quality, with the remaining 7 low quality. The most common score was a 1 which generally indicated that papers covered the method, but with insufficient detail or justification. The stand-out anomaly was the lack of sample size justification, where 42 (80.8%) papers scored 0, in line with previous reports of this being an issue in ML research in RA.^58^ There was no correlation between the quality of the papers and their consideration of sex bias (r=0.04, p=0.76), with the only high-quality paper scoring 0 for sex bias consideration.^58^

## Discussion

We observed a substantial body of literature from EMBASE and PubMed pertaining to ML applied in RA. We applied a specifically developed sex bias checking matrix to the 52 papers that passed our screening criteria. Of these, 42 research papers did not report or consider the potential for sex bias in any of our five categories: training data, test data, input variables, output variables or analysis. Only one reported potential sex bias in input variables, six in the output variables, while three disaggregated their model performance results by sex. No paper attempted to mitigate potential sex biases in their model development process. Our analysis demonstrates that sex bias is generally not considered in the development of ML models and raises important questions about how relevant the results of these studies are for clinical application and their lack of contribution to the mitigation of sex bias in healthcare.

Analysis bias, which involves assessing model performance by sex, rather than using a composite metric, is arguably the most impactful of the five types of bias we assessed. It is essential for demonstrating the presence and direction of bias and allows us to understand the extent of impact, if any, of data skews or biased model variables, as well as the efficacy of mitigation methods. Only 3 papers (Bai *et al*, Morales-Ivorra *et al* and Kalweit *et al*) of the 52 reviewed disaggregated model performance by sex, though the differences were generally not tested statistically, and minimal explanation was attached to their findings.^57 59 60^

A major finding is that no paper commented on the potential impacts of sex skews in data, despite a mean sex split of 74% female participants across included papers. This information was readily available to the authors as papers had to provide the sex split of RA patients to be included in our review. One likely explanation for the dearth of data bias reporting is that most researchers considered their female-skewed data to reflect the sex bias in RA prevalence in the general population (3:1 females: males), thereby mirroring food and drug administation (FDA) guidance for clinical trials (particularly as ‘predicting treatment response’ was the most common objective).^61^ Regardless, it is known that sex imbalance in data generally makes results less applicable to the minority groups, a discrepancy which can be further exacerbated in ML research.^19 62^ Furthermore, it is noteworthy that 1 paper contained a dataset in which only 17% of their 291 RA participants were female,^48^ in sharp contrast with the reported sex prevalence. Additionally, three other papers relied on datasets with an overwhelming female representation (over 85%).^63^,^65^ The absence of acknowledgement of the impact of skewed sex distributions on the representativeness of algorithmic predictions within these papers, despite easily implementable data balancing mitigation methods,^19 62 66 67^ suggests an element of limited awareness or underprioritisation of fair and equitable models.

While one paper assessed and reported no sex difference in the model’s input variables, six papers recognised potential sex bias in their output variables, specifically regarding a skew to higher scores in females on the disease activity measures. This observation has been previously reported in the RA literature and is believed to be rooted in the self-report nature of the composite measure, rather than the physical or physiological aspects it encompasses.^38^ The inclination for women to report higher pain scores has been extensively documented, yet the underlying causes remain unclear. It is possible that this divergence in self-reported scores arises from a combination of poorly understood physiological and psychological factors.^68 69^ The challenge lies in distinguishing between sex bias in measurement and genuine sex-based differences in disease activity within the general population.

Mitigating bias in ML models has been well researched in the literature, with several strategies including the use of diverse and representative datasets, reweighting models and employing advanced tools such as the Health Equity Assessment Toolkit^70^ and fairness detector libraries like AIF360.^71^ Techniques such as resampling, adjusting decision thresholds for different sex groups, stratified cross-validation and creating sex-specific models are well documented in the literature and readily available.^72^ While some techniques are available and effective, using balanced data or incorporating representative variables is often the most effective approach for simultaneously maintaining model performance, and as such has been the focus of our review.^73^,^75^ Despite the availability of these mitigation strategies, the primary challenge lies in raising awareness and ensuring their widespread adoption among researchers. To address this issue, the implementation of standardised guidelines is crucial. The ‘Sex and Gender Equity in Research’ guidelines^76^ provide a comprehensive framework for reporting sex and gender considerations in research, while the ‘Guidelines for Accurate and Transparent Health Estimates Reporting’^77^ offer valuable recommendations for ensuring transparency and accuracy in health research. These guidelines, endorsed by leading organisations, can serve as essential tools for researchers to systematically address sex bias in their studies.

Several limitations of the research methods and results are acknowledged, many of which are inherent to systematic reviews. Publication bias means that we may not have captured all available studies in the field. The search strategy, while comprehensive, relies on the specific terms and keywords used. As a result, it may miss relevant studies due to differences in terminology, such as synonyms and the way concepts are described across studies. Additionally, the review was limited to papers published in the English language, thus excluding relevant non-English studies. The diversity in study methods, measures and study populations across the papers we reviewed introduced significant heterogeneity, making it challenging to apply our inclusion/exclusion criteria consistently across the entire dataset. Similarly, the types of bias assessed were not complete^78^ but enabled consistency in our review. Another key limitation pertains to the limited explanation of the collection and representation of sex and gender features within almost all papers included in our analysis, potentially leading to conflation of these two distinct concepts.

While this study aimed to shed light on the specific issue of sex bias in the context of RA, a comprehensive assessment of bias in healthcare algorithms demands a broader perspective. For instance, conditions such as cardiovascular disease, diabetes and cancer also exhibit sex differences in prevalence and outcomes, which could similarly influence the presence and impact of gender bias in ML models. Notably, women are often under-represented in clinical trials and research datasets, potentially leading to biases in diagnostic and treatment algorithms even in areas without sex-specific differences. Moreover, existing literature suggests biases in ML have negative impacts on other underrepresented or minority groups, including racial and ethnic minorities.^79 80^ Increased consideration of the types of bias outlined here is imperative to ensuring the inclusion of diverse and representative data is critical for the development of equitable and accurate models to enhance the fairness and reliability of ML applications in healthcare.

## supplementary material

10.1136/bmjopen-2024-086117 online supplemental file 1

## Data Availability

All data relevant to the study are included in the article or uploaded as supplementary information.
