# Supplementary material for: Sex bias consideration in healthcare machine-learning research: a systematic review in rheumatoid arthritis
Source: BMJ Open. 2025 Mar 13;15(3):e086117. doi: 10.1136/bmjopen-2024-086117 (PMC11906982; doi:10.1136/bmjopen-2024-086117)
Supplement: online supplemental file 1 [file bmjopen-15-3-s001.pdf]

Public

No.	First Author	Title	Year	Study Objective	ML Models	Overall Sex Split % Female RA	Training Data Female RA %	Test Data Female RA %	Training data Bias	Test Data Bias	Input Variable Bias	Output Variable Bias	Analysis Bias	Total (X/10)	Pre-processing	Cross-validation	Sample Size	Evaluation	Hyperparameters	Reproducibility	Total (X/12)	Reference
1	Bai	Improved diagnosis of rheumatoid arthritis using an artificial neural network	2022	Improve Diagnostic Accuracy	NN	17	NA	NA	0	0	0	0	1	1	1	1	0	1	2	0	5	Bai, L., Zhang, Y., Wang, P., Zhu, X., Xiong, J. W., & Cui, L. (2022). Improved diagnosis of rheumatoid arthritis using an artificial neural network. Scientific Reports, 12(1). https://doi.org/10.1038/S41598-022-13750-9
2	Bouget	Machine learning predicts response to TNF inhibitors in rheumatoid arthritis: results on the ESPOIR and ABIRISK cohorts	2022	Predict Treatment Response	Linear Regression, Random Forest, Boosted Tree	71	71	72	0	0	0	0	0	0	1	2	0	2	0	1	6	Bouget, V., Duquesne, J., Hassler, S., Cournède, P.-H., Fautrel, B., Guillemin, F., Pallardy, M., Broët, P., Mariette, X., & Bitoun, S. (2022). Machine learning predicts response to TNF inhibitors in rheumatoid arthritis: results on the ESPOIR and ABIRISK cohorts. RMD Open, 8(2). https://doi.org/10.1136/rmdopen-2022-002442
3	Chin	EDram: Effective early disease risk assessment with matrix factorization on a large-scale medical database: A case study on rheumatoid arthritis	2018	Improve Diagnostic Accuracy	SVM	76	NA	NA	0	0	0	0	0	0	2	1	0	1	2	1	7	Chin, C.-Y., Hsieh, S.-Y., & Tseng, V. S. (2018). EDram: Effective early disease risk assessment with matrix factorization on a large-scale medical database: A case study on rheumatoid arthritis. PLoS ONE, 13(11). https://doi.org/10.1371/journal.pone.0207579
4	Chocholova	Glycomics meets artificial intelligence – Potential of glycan analysis for identification of seropositive and seronegative rheumatoid arthritis patients revealed	2018	Identify Patient Subgroups	NN	83	NA	NA	0	0	0	0	0	0	2	1	0	0	1	0	4	Chocholova, E., Bertok, T., Jane, E., Lorencova, L., Holazova, A., Belicka, L., Belicky, S., Mislovicova, D., Vikartovska, A., Imrich, R., Kasak, P., & Tkac, J. (2018). Glycomics meets artificial intelligence – Potential of glycan analysis for identification of seropositive and seronegative rheumatoid arthritis patients revealed. Clinica Chimica Acta, 481, 49–55. https://doi.org/10.1016/j.cca.2018.02.031
5	Christensen	Applying cascaded convolutional neural network design further enhances automatic scoring of arthritis disease activity on ultrasound images from rheumatoid arthritis patients	2020	Score Disease Activity	NN	68	NA	NA	0	0	0	0	0	0	2	1	0	0	0	0	3	Christensen, A. B. H., Just, S. A., Andersen, J. K. H., & Savarimuthu, T. R. (2020). Applying cascaded convolutional neural network design further enhances automatic scoring of arthritis disease activity on ultrasound images from rheumatoid arthritis patients. Annals of the Rheumatic Diseases, 79(9), 1189–1193. https://doi.org/10.1136/annrheumdis-2019-216636
6	Cuppen	Proteomics to predict the response to tumour necrosis factor-α inhibitors in rheumatoid arthritis using a supervised cluster-analysis based protein score	2018	Predict Treatment Response	Logistic Regression	74	72	75	0	0	0	0	0	0	2	1	0	2	0	1	6	Cuppen, B. V. J., Fritsch-Stork, R. D. E., Eekhout, I., de Jager, W., Marijnissen, A. C., Bijlsma, J. W. J., Custers, M., van Laar, J. M., Lafeber, F. P. J. G., & Welsing, P. M. J. (2018). Proteomics to predict the response to tumour necrosis factor-α inhibitors in rheumatoid arthritis using a supervised cluster-analysis based protein score. Scandinavian Journal of Rheumatology, 47(1), 12–21. https://doi.org/10.1080/03009742.2017.1309061
7	Curtis	Machine Learning Applied to Patient-Reported Outcomes to Classify Physician-Derived Measures of Rheumatoid Arthritis Disease Activity	2022	Score Disease Activity	Random Forest, Boosted Tree, SVM, Logistic Regression	83	NA	NA	0	0	0	1	0	1	1	1	0	1	1	1	5	Curtis, J. R., Su, Y., Black, S., Xu, S., Langholff, W., Bingham, C. O., Kafka, S., & Xie, F. (2022). Machine Learning Applied to Patient-Reported Outcomes to Classify Physician-Derived Measures of Rheumatoid Arthritis Disease Activity. ACR Open Rheumatology, 4(12), 995–1003. https://doi.org/10.1002/acr2.11499
8	de la Calle-Fabregat	Prediction of the Progression of Undifferentiated Arthritis to Rheumatoid Arthritis Using DNA Methylation Profiling	2021	Improve Diagnostic Accuracy	Logistic Regression, Random Forest, SVM	57	58	56	0	0	0	0	0	0	2	2	0	1	0	1	6	de la Calle-Fabregat, C., Niemantsverdriet, E., Cañete, J. D., Li, T., van der Helm-van Mil, A. H. M., Rodríguez-Ubreva, J., & Ballestar, E. (2021). Prediction of the Progression of Undifferentiated Arthritis to Rheumatoid Arthritis Using DNA Methylation Profiling. Arthritis and Rheumatology, 73(12), 2229–2239. https://doi.org/10.1002/art.41885
9	Duong	Clinical predictors of response to methotrexate in patients with rheumatoid arthritis: a machine learning approach using clinical trial data	2022	Predict Treatment Response	Logistic Regression, Random Forest	80	78	81	0	0	0	1	0	1	1	2	1	2	0	1	7	Duong, S. Q., Crowson, C. S., Athreya, A., Atkinson, E. J., Davis, J. M., Warrington, K. J., Matteson, E. L., Weinshilboum, R., Wang, L., & Myasoedova, E. (2022). Clinical predictors of response to methotrexate in patients with rheumatoid arthritis: a machine learning approach using clinical trial data. Arthritis Research and Therapy, 24(1). https://doi.org/10.1186/s13075-022-02851-5
10	Duquesne	Machine learning identifies a profile of inadequate responder to methotrexate in rheumatoid arthritis	2022	Predict Treatment Response	Logistic Regression, Random Forest, Boosted Tree	71	70	72	0	0	0	0	0	0	2	2	1	2	0	1	8	Duquesne, J., Bouget, V., Cournède, P. H., Fautrel, B., Guillemin, F., de Jong, P. H. P., Heutz, J. W., Verstappen, M., van der Helm-van Mil, A. H. M., Mariette, X., & Bitoun, S. (2022). Machine learning identifies a profile of inadequate responder to methotrexate in rheumatoid arthritis. Rheumatology (Oxford, England). https://doi.org/10.1093/rheumatology/keac645
11	Feldman	Supplementing Claims Data with Electronic Medical Records to Improve Estimation and Classification of Rheumatoid Arthritis Disease Activity: A Machine Learning Approach	2019	Score Disease Activity	Linear Regression, Logistic Regression	80	NA	NA	0	0	0	0	0	0	1	1	0	1	2	1	6	Feldman, C. H., Yoshida, K., Xu, C., Frits, M. L., Shadick, N. A., Weinblatt, M. E., Connolly, S. E., Alemao, E., & Solomon, D. H. (2019). Supplementing Claims Data with Electronic Medical Records to Improve Estimation and Classification of Rheumatoid Arthritis Disease Activity: A Machine Learning Approach. ACR Open Rheumatology, 1(9), 552–559. https://doi.org/10.1002/acr2.11068

Public

12	Folle	Deep Learning-Based Classification of Inflammatory Arthritis by Identification of Joint Shape Patterns—How Neural Networks Can Tell Us Where to "Deep Dive" Clinically	2022	Improve Diagnostic Accuracy	NN	69	NA	NA	0	0	0	0	0	0	2	1	0	1	0	1	5	Folle, L., Bayat, S., Kleyer, A., Fagni, F., Kapsner, L. A., Schlereth, M., Meinderink, T., Breininger, K., Tascilar, K., Krönke, G., Uder, M., Sticherling, M., Bickelhaupt, S., Schett, G., Maier, A., Roemer, F., & Simon, D. (2022). Advanced neural networks for classification of MRI in psoriatic arthritis, seronegative, and seropositive rheumatoid arthritis. Rheumatology (Oxford, England), 61(12), 4945–4951. https://doi.org/10.1093/RHEUMATOLOGY/KEAC197
13	Folle	Advanced neural networks for classification of MRI in psoriatic arthritis, seronegative, and seropositive rheumatoid arthritis	2022	Identify Patient Subgroups	NN	67	NA	NA	0	0	0	0	0	0	0	1	0	1	1	2	5	Folle, L., Simon, D., Tascilar, K., Krönke, G., Liphardt, A.-M., Maier, A., Schett, G., & Kleyer, A. (2022). Deep Learning-Based Classification of Inflammatory Arthritis by Identification of Joint Shape Patterns—How Neural Networks Can Tell Us Where to "Deep Dive" Clinically. Frontiers in Medicine, 9. https://doi.org/10.3389/fmed.2022.850552
14	Fukae	Convolutional neural network for classification of two-dimensional array images generated from clinical information may support diagnosis of rheumatoid arthritis	2020	Improve Diagnostic Accuracy	NN	60	60	60	0	0	0	0	0	0	1	1	0	0	1	0	3	Fukae, J., Isobe, M., Hattori, T., Fujieda, Y., Kono, M., Abe, N., Kitano, A., Narita, A., Henmi, M., Sakamoto, F., Aoki, Y., Ito, T., Mitsuzaki, A., Matsuhashi, M., Shimizu, M., Tanimura, K., Sutherland, K., Kamishima, T., Atsumi, T., & Koike, T. (2020). Convolutional neural network for classification of two-dimensional array images generated from clinical information may support diagnosis of rheumatoid arthritis. Scientific Reports, 10(1), 5648. https://doi.org/10.1038/s41598-020-62634-3
15	Geng	Prediction of diagnosis results of rheumatoid arthritis patients based on autoantibodies and cost-sensitive neural network	2022	Improve Diagnostic Accuracy	NN	78	NA	NA	0	0	0	0	0	0	1	1	0	0	1	1	4	Geng, L., Qu, W., Wang, S., Chen, J., Xu, Y., Kong, W., Xu, X., Feng, X., Zhao, C., Liang, J., Zhang, H., & Sun, L. (2022). Prediction of diagnosis results of rheumatoid arthritis patients based on autoantibodies and cost-sensitive neural network. Clinical Rheumatology, 41(8), 2329–2339. https://doi.org/10.1007/s10067-022-06109-y
16	Gossec	Detection of Flares by Decrease in Physical Activity, Collected Using Wearable Activity Trackers in Rheumatoid Arthritis or Axial Spondyloarthritis: An Application of Machine Learning Analyses in Rheumatology	2019	Score Disease Activity	Naïve Bayes	83	NA	NA	0	0	0	0	0	0	2	1	0	1	0	1	5	Gossec, L., Guyard, F., Leroy, D., Lafargue, T., Seiler, M., Jacquemin, C., Molto, A., Sellam, J., Foltz, V., Gandjbakhch, F., Hudry, C., Mitrovic, S., Fautrel, B., & Servy, H. (2019). Detection of Flares by Decrease in Physical Activity, Collected Using Wearable Activity Trackers in Rheumatoid Arthritis or Axial Spondyloarthritis: An Application of Machine Learning Analyses in Rheumatology. Arthritis Care and Research, 71(10), 1336–1343. https://doi.org/10.1002/acr.23768
17	Gosselt	Complex machine-learning algorithms and multivariable logistic regression on par in the prediction of insufficient clinical response to methotrexate in rheumatoid arthritis	2021	Predict Treatment Response	Logistic Regression, Random Forest, Boosted Tree	68	NA	NA	0	0	0	0	0	0	1	1	2	2	1	1	8	Gosselt, H. R., Verhoeven, M. M. A., Bulatović-ćalasan, M., Welsing, P. M., de Rotte, M. C. F. J., Hazes, J. M. W., Lafeber, F. P. J. G., Hoogendoorn, M., & de Jonge, R. (2021). Complex machine-learning algorithms and multivariable logistic regression on par in the prediction of insufficient clinical response to methotrexate in rheumatoid arthritis. Journal of Personalized Medicine, 11(1), 1–12. https://doi.org/10.3390/jpm11010044
18	Guan	Machine Learning to Predict Anti–Tumor Necrosis Factor Drug Responses of Rheumatoid Arthritis Patients by Integrating Clinical and Genetic Markers	2019	Predict Treatment Response	Random Forest, Boosted Tree, Linear Regression, Logistic Regression, SVM, Other	76	75	78	0	0	0	0	0	0	0	2	0	1	2	1	6	Guan, Y., Zhang, H., Quang, D., Wang, Z., Parker, S. C. J., Pappas, D. A., Kremer, J. M., & Zhu, F. (2019). Machine Learning to Predict Anti–Tumor Necrosis Factor Drug Responses of Rheumatoid Arthritis Patients by Integrating Clinical and Genetic Markers. Arthritis and Rheumatology, 71(12), 1987–1996. https://doi.org/10.1002/art.41056
19	Hirano	Development and validation of a deep-learning model for scoring of radiographic finger joint destruction in rheumatoid arthritis	2019	Assess Joint Damage	NN	83	83	87	0	0	0	0	0	0	1	1	0	0	1	0	3	Hirano, T., Nishide, M., Nonaka, N., Seita, J., Ebina, K., Sakurada, K., & Kumanogoh, A. (2019). Development and validation of a deep-learning model for scoring of radiographic finger joint destruction in rheumatoid arthritis. Rheumatology Advances in Practice, 3(2). https://doi.org/10.1093/rap/rkz047
20	Honda	Development of a scoring model for the Sharp/van der Heijde score using convolutional neural networks and its clinical application	2022	Assess Joint Damage	NN, Random Forest	89	NA	NA	0	0	0	0	0	0	2	1	1	1	1	1	7	Honda, S., Yano, K., Tanaka, E., Ikari, K., & Harigai, M. (2022). Development of a scoring model for the Sharp/van der Heijde score using convolutional neural networks and its clinical application. Rheumatology (Oxford, England). https://doi.org/10.1093/rheumatology/keac586
21	Johansson	Predicting response to tocilizumab monotherapy in rheumatoid arthritis: A real-world data analysis using machine learning	2021	Predict Treatment Response	Logistic Regression, Random Forest	76	NA	NA	0	0	0	0	0	0	1	1	0	2	0	0	4	Johansson, F. D., Collins, J. E., Yau, V., Guan, H., Kim, S. C., Losina, E., Sontag, D., Stratton, J., Trinh, H., Greenberg, J., & Solomon, D. H. (2021). Predicting response to tocilizumab monotherapy in rheumatoid arthritis: A real-world data analysis using machine learning. Journal of Rheumatology, 48(9), 1364–1370. https://doi.org/10.3899/jrheum.201626

Public

22	Kalweit	Personalized prediction of disease activity in patients with rheumatoid arthritis using an adaptive deep neural network	2021	Score Disease Activity	NN, Random Forest, Linear Regression, Logistic Regression, SVM	74	NA	NA	0	0	0	0	1	1	1	1	0	1	1	1	5	Kalweit, M., Walker, U. A., Finckh, A., Müller, R., Kalweit, G., Scherer, A., Boedecker, J., & Hügle, T. (2021). Personalized prediction of disease activity in patients with rheumatoid arthritis using an adaptive deep neural network. PLoS ONE, 16(6 June). https://doi.org/10.1371/journal.pone.0252289
23	Kim	Effects of RETN polymorphisms on treatment response in rheumatoid arthritis patients receiving TNF-α inhibitors and utilization of machine-learning algorithms	2022	Predict Treatment Response	Logistic Regression, Random Forest, SVM	81	NA	NA	0	0	0	0	0	0	0	1	0	1	2	1	5	Kim, W., Jin Oh, S., Thi Trinh, N., Yeon Gil, J., Ah Choi, I., Hyoun Kim, J., Hee Kim, J., Jung, J.-Y., Kim, J., Kim, H.-A., & Eun Lee, K. (2022). Effects of RETN polymorphisms on treatment response in rheumatoid arthritis patients receiving TNF-α inhibitors and utilization of machine-learning algorithms. International Immunopharmacology, 111. https://doi.org/10.1016/j.intimp.2022.109094
24	Kim	Association of TLR 9 gene polymorphisms with remission in patients with rheumatoid arthritis receiving TNF-α inhibitors and development of machine learning models	2021	Predict Treatment Response	Random Forest, SVM, Logistic Regression	81	NA	NA	0	0	0	0	0	0	2	1	0	1	2	1	7	Kim, W., Kim, T. H., Oh, S. J., Kim, H. J., Kim, J. H., Kim, H.-A., Jung, J.-Y., Choi, I. A., & Lee, K. E. (2021). Association of TLR 9 gene polymorphisms with remission in patients with rheumatoid arthritis receiving TNF-α inhibitors and development of machine learning models. Scientific Reports, 11(1), 20169. https://doi.org/10.1038/s41598-021-99625-x
25	Kim	Association between SYVN1 and SEL1 genetic polymorphisms and remission in rheumatoid arthritis patients treated with TNF-α inhibitors: a machine learning approach	2023	Predict Treatment Response	Logistic Regression, Random Forest, SVM	81	NA	NA	0	0	0	0	0	0	2	1	0	1	2	1	7	Kim, W., Yeon, H. R., Kim, J. H., Kim, J. H., Kim, J. H., Kim, H.-A., Jung, J.-Y., Kim, J., Choi, I. A., & Lee, K. E. (2023). Association between SYVN1 and SEL1 genetic polymorphisms and remission in rheumatoid arthritis patients treated with TNF-α inhibitors: a machine learning approach. Immunologic Research. https://doi.org/10.1007/s12026-023-09382-4
26	Koo	Machine learning model for identifying important clinical features for predicting remission in patients with rheumatoid arthritis treated with biologics	2021	Predict Treatment Response	Logistic Regression, SVM, Random Forest, Boosted Tree	83	NA	NA	0	0	0	0	0	0	1	1	0	0	2	1	5	Koo, B. S., Eun, S., Shin, K., Yoon, H., Hong, C., Kim, D.-H., Hong, S., Kim, Y.-G., Lee, C.-K., Yoo, B., & Oh, J. S. (2021). Machine learning model for identifying important clinical features for predicting remission in patients with rheumatoid arthritis treated with biologics. Arthritis Research and Therapy, 23(1). https://doi.org/10.1186/s13075-021-02567-y
27	Lee	Machine learning-based prediction model for responses of bDMARDs in patients with rheumatoid arthritis and ankylosing spondylitis	2021	Predict Treatment Response	Random Forest, Boosted Tree, SVM, NN	84	NA	NA	0	0	0	0	0	0	0	2	0	1	2	2	7	Lee, S., Kang, S., Eun, Y., Won, H.-H., Kim, H., Lee, J., Koh, E.-M., & Cha, H.-S. (2021). Machine learning-based prediction model for responses of bDMARDs in patients with rheumatoid arthritis and ankylosing spondylitis. Arthritis Research and Therapy, 23(1). https://doi.org/10.1186/s13075-021-02635-3
28	Lim	Functional coding haplotypes and machine-learning feature elimination identifies predictors of Methotrexate Response in Rheumatoid Arthritis patients	2022	Predict Treatment Response	NN, SVM, Logistic Regression, Random Forest, Boosted Tree	82	NA	NA	0	0	0	0	0	0	1	1	0	1	1	1	5	Lim, A. J. W., Lim, L. J., Ooi, B. N. S., Koh, E. T., Tan, J. W. L., Chong, S. S., Khor, C. C., Tucker-Kellogg, L., Leong, K. P., & Lee, C. G. (2022). Functional coding haplotypes and machine-learning feature elimination identifies predictors of Methotrexate Response in Rheumatoid Arthritis patients. EBioMedicine, 75. https://doi.org/10.1016/j.ebiom.2021.103800
29	Lim	Machine learning using genetic and clinical data identifies a signature that robustly predicts methotrexate response in rheumatoid arthritis	2022	Predict Treatment Response	SVM, NN, Random Forest, Boosted Tree, Logistic Regression	82	82	84	0	0	0	0	0	0	2	1	0	1	1	1	6	Lim, L. J., Lim, A. J. W., Ooi, B. N. S., Tan, J. W. L., Koh, E. T., Chong, S. S., Khor, C. C., Tucker-Kellogg, L., Lee, C. G., & Leong, K. P. (2022). Machine learning using genetic and clinical data identifies a signature that robustly predicts methotrexate response in rheumatoid arthritis. Rheumatology (United Kingdom), 61(10), 4175–4186. https://doi.org/10.1093/rheumatology/keac032
30	Lötsch	Machine-learning-based knowledge discovery in rheumatoid arthritis-related registry data to identify predictors of persistent pain	2020	Identify Patient Subgroups	Random Forest, KNN, SVM, Naïve Bayes, Boosted Tree, NN	73	NA	NA	0	0	0	0	0	0	2	1	1	0	2	1	7	Lötsch, J., Alfredsson, L., & Lampa, J. (2020). Machine-learning-based knowledge discovery in rheumatoid arthritis-related registry data to identify predictors of persistent pain. Pain, 161(1), 114–126. https://doi.org/10.1097/j.pain.0000000000001693
31	Maciejewski	Prediction of response of methotrexate in patients with rheumatoid arthritis using serum lipidomics	2021	Predict Treatment Response	SVM, Random Forest, Linear Regression, Logistic Regression	80	NA	NA	0	0	0	0	0	0	1	1	0	1	2	1	6	Maciejewski, M., Sands, C., Nair, N., Ling, S., Verstappen, S., Hyrich, K., Barton, A., Ziemek, D., Lewis, M. R., & Plant, D. (2021). Prediction of response of methotrexate in patients with rheumatoid arthritis using serum lipidomics. Scientific Reports, 11(1), 7266. https://doi.org/10.1038/s41598-021-86729-7

Public

32	Matsuo	Machine learning-based prediction of relapse in rheumatoid arthritis patients using data on ultrasound examination and blood test	2022	Predict Treatment Response	Logistic Regression, Random Forest, Boosted Tree	82	NA	NA	0	0	0	0	0	0	1	1	0	1	2	0	5	Matsuo, H., Kamada, M., Imamura, A., Shimizu, M., Inagaki, M., Tsuji, Y., Hashimoto, M., Tanaka, M., Ito, H., & Fujii, Y. (2022). Machine learning-based prediction of relapse in rheumatoid arthritis patients using data on ultrasound examination and blood test. Scientific Reports, 12(1), 7224. https://doi.org/10.1038/s41598-022-11361-y
33	Mehta	Machine learning identification of thresholds to discriminate osteoarthritis and rheumatoid arthritis synovial inflammation	2023	Improve Diagnostic Accuracy	Random Forest	83	NA	NA	0	0	0	0	0	0	1	1	0	1	2	1	6	Mehta, B., Goodman, S., DiCarlo, E., Jannat-Khah, D., Gibbons, J. A. B., Otero, M., Donlin, L., Pannellini, T., Robinson, W. H., Sculco, P., Figgie, M., Rodriguez, J., Kirschmann, J. M., Thompson, J., Slater, D., Frezza, D., Xu, Z., Wang, F., & Orange, D. E. (2023). Machine learning identification of thresholds to discriminate osteoarthritis and rheumatoid arthritis synovial inflammation. Arthritis Research and Therapy, 25(1). https://doi.org/10.1186/s13075-023-03008-8
34	Morales-Ivorra	Assessment of inflammation in patients with rheumatoid arthritis using thermography and machine learning: A fast and automated technique	2022	Score Disease Activity	KNN	77	75	80	0	0	0	0	1	1	0	1	1	1	1	1	5	Morales-Ivorra, I., Narváez, J., Gómez-Vaquero, C., Moragues, C., Nolla, J. M., Narváez, J. A., & Marín-López, M. A. (2022). Assessment of inflammation in patients with rheumatoid arthritis using thermography and machine learning: A fast and automated technique. RMD Open, 8(2). https://doi.org/10.1136/rmdopen-2022-002458
35	Myasoedova	Toward Individualized Prediction of Response to Methotrexate in Early Rheumatoid Arthritis: A Pharmacogenomics-Driven Machine Learning Approach	2022	Predict Treatment Response	Random Forest	71	71	71	0	0	0	1	0	1	1	2	0	2	2	0	7	Myasoedova, E., Athreya, A. P., Crowson, C. S., Davis, J. M., Warrington, K. J., Walchak, R. C., Carlson, E., Kalari, K. R., Bongartz, T., Tak, P. P., van Vollenhoven, R. F., Padyukov, L., Emery, P., Morgan, A., Wang, L., Weinshilboum, R. M., & Matteson, E. L. (2022). Toward Individualized Prediction of Response to Methotrexate in Early Rheumatoid Arthritis: A Pharmacogenomics-Driven Machine Learning Approach. Arthritis Care and Research, 74(6), 879–888. https://doi.org/10.1002/acr.24834
36	Norgeot	Assessment of a Deep Learning Model Based on Electronic Health Record Data to Forecast Clinical Outcomes in Patients With Rheumatoid Arthritis	2019	Score Disease Activity	NN	NA	82	NA	0	0	0	0	0	0	2	2	1	1	2	1	9	Norgeot, B., Glicksberg, B. S., Trupin, L., Lituiev, D., Gianfrancesco, M., Oskotsky, B., Schmajuk, G., Yazdany, J., & Butte, A. J. (2019). Assessment of a Deep Learning Model Based on Electronic Health Record Data to Forecast Clinical Outcomes in Patients With Rheumatoid Arthritis. JAMA Network Open, 2(3), e190606. https://doi.org/10.1001/jamanetworkopen.2019.0606
37	Pauk	A computational method to differentiate rheumatoid arthritis patients using thermography data	2022	Improve Diagnostic Accuracy	NN	84	NA	NA	0	0	0	0	0	0	1	1	0	0	1	0	3	Pauk, J., Trinkunas, J., Puronaite, R., Ihnatouski, M., & Wasilewska, A. (2022). A computational method to differentiate rheumatoid arthritis patients using thermography data. Technology and Health Care, 30(1), 209–216. https://doi.org/10.3233/THC-219004
38	Prasad	ATRPred: A machine learning based tool for clinical decision making of anti-TNF treatment in rheumatoid arthritis patients	2022	Predict Treatment Response	KNN	76	NA	NA	0	0	0	1	0	1	2	1	0	1	1	2	7	Prasad, B., McGeough, C., Eakin, A., Ahmed, T., Small, D., Gardiner, P., Pendleton, A., Wright, G., Bjourson, A. J., Gibson, D. S., & Shukla, P. (2022). ATRPred: A machine learning based tool for clinical decision making of anti-TNF treatment in rheumatoid arthritis patients. PLoS Computational Biology, 18(7). https://doi.org/10.1371/journal.pcbi.1010204
39	Radke	Adaptive IoU Thresholding for Improving Small Object Detection: A Proof-of-Concept Study of Hand Erosions Classification of Patients with Rheumatic Arthritis on X-ray Images	2023	Assess Joint Damage	NN	67	65	75	0	0	0	0	0	0	2	1	0	1	1	1	6	Radke, K. L., Kors, M., Müller-Lutz, A., Frenken, M., Wilms, L. M., Baraliakos, X., Wittsack, H.-J., Distler, J. H. W., Abrar, D. B., Antoch, G., & Sewerin, P. (2023). Adaptive IoU Thresholding for Improving Small Object Detection: A Proof-of-Concept Study of Hand Erosions Classification of Patients with Rheumatic Arthritis on X-ray Images. Diagnostics, 13(1). https://doi.org/10.3390/diagnostics13010104
40	Reed	Pilot study of a machine-learning tool to assist in the diagnosis of hand arthritis	2022	Improve Diagnostic Accuracy	Random Forest, Logistic Regression, SVM	68	NA	NA	0	0	0	0	0	0	1	1	0	0	0	0	2	Reed, M., Le Souëf, T., & Rampono, E. (2022). Pilot study of a machine-learning tool to assist in the diagnosis of hand arthritis. Internal Medicine Journal, 52(6), 959–967. https://doi.org/10.1111/imj.15173
41	Srinivasan	A Framework of Faster CRNN and VGG16-Enhanced Region Proposal Network for Detection and Grade Classification of Knee RA	2023	Assess Joint Damage	NN	60	NA	NA	0	0	0	0	0	0	1	1	0	0	1	0	3	Srinivasan, S., Gunasekaran, S., Mathivanan, S. K., Jayagopal, P., Khan, M. A., Alasiry, A., Marzougui, M., & Masood, A. (2023). A Framework of Faster CRNN and VGG16-Enhanced Region Proposal Network for Detection and Grade Classification of Knee RA. Diagnostics, 13(8). https://doi.org/10.3390/diagnostics13081385
42	Tao	Multiomics and Machine Learning Accurately Predict Clinical Response to Adalimumab and Etanercept Therapy in Patients With Rheumatoid Arthritis	2021	Predict Treatment Response	Random Forest	70	NA	NA	0	0	0	1	0	1	2	2	0	1	2	1	8	Tao, W., Concepcion, A. N., Vianen, M., Marijnissen, A. C. A., Lafeber, F. P. G. J., Radstake, T. R. D. J., & Pandit, A. (2021). Multiomics and Machine Learning Accurately Predict Clinical Response to Adalimumab and Etanercept Therapy in Patients With Rheumatoid Arthritis. Arthritis and Rheumatology, 73(2), 212–222. https://doi.org/10.1002/art.41516
43	Venerito	A Machine Learning Approach for Predicting Sustained Remission in Rheumatoid Arthritis Patients on Biologic Agents	2022	Predict Treatment Response	Logistic Regression, Random Forest, Boosted Tree, KNN	88	NA	NA	0	0	0	0	0	0	1	1	2	2	2	0	8	Venerito, V., Angelini, O., Fornaro, M., Cacciapaglia, F., Lopalco, G., & Iannone, F. (2022). A Machine Learning Approach for Predicting Sustained Remission in Rheumatoid Arthritis Patients on Biologic Agents. Journal of Clinical Rheumatology, 28(2), E334–E339. https://doi.org/10.1097/RHU.0000000000001720

Public

44	Vodencarevic	Advanced machine learning for predicting individual risk of flares in rheumatoid arthritis patients tapering biologic drugs	2021	Predict Treatment Response	Logistic Regression, KNN, Naïve Bayes, Random Forest	59	NA	NA	0	0	0	0	0	0	1	1	0	2	2	1	7	Vodencarevic, A., Tascilar, K., Hartmann, F., Reiser, M., Hueber, A. J., Haschka, J., Bayat, S., Meinderink, T., Knitza, J., Mendez, L., Hagen, M., Krönke, G., Rech, J., Manger, B., Kleyer, A., Zimmermann-Rittereiser, M., Schett, G., & Simon, D. (2021). Advanced machine learning for predicting individual risk of flares in rheumatoid arthritis patients tapering biologic drugs. Arthritis Research and Therapy, 23(1). https://doi.org/10.1186/s13075-021-02439-5
45	Zhou	RATING: Medical knowledge-guided rheumatoid arthritis assessment from multimodal ultrasound images via deep learning	2022	Score Disease Activity	NN	70	70	69	0	0	0	0	0	0	2	2	0	1	1	2	8	Zhou, Z., Zhao, C., Qiao, H., Wang, M., Guo, Y., Wang, Q., Zhang, R., Wu, H., Dong, F., Qi, Z., Li, J., Tian, X., Zeng, X., Jiang, Y., Xu, F., Dai, Q., & Yang, M. (2022). RATING: Medical knowledge-guided rheumatoid arthritis assessment from multimodal ultrasound images via deep learning. Patterns, 3(10). https://doi.org/10.1016/J.PATTER.2022.100592
46	Plant	Profiling of Gene Expression Biomarkers as a Classifier of Methotrexate Nonresponse in Patients With Rheumatoid Arthritis	2019	Predict Treatment Response	Logistic Regression, Random Forest, Other	77	NA	NA	0	0	0	0	0	0	1	1	1	1	1	1	6	Plant, D., Maciejewski, M., Smith, S., Nair, N., Hyrich, K., Ziemek, D., Barton, A., & Verstappen, S. (2019). Profiling of Gene Expression Biomarkers as a Classifier of Methotrexate Nonresponse in Patients With Rheumatoid Arthritis. Arthritis & Rheumatology (Hoboken, N.J.), 71(5), 678–684. https://doi.org/10.1002/ART.40810
47	Wu	Metagenomics Biomarkers Selected for Prediction of Three Different Diseases in Chinese Population	2018	Score Disease Activity	KNN, Logistic Regression, Random Forest, SVM, Boosted Tree	51	NA	NA	0	0	0	0	0	0	1	1	0	1	0	1	4	Wu, H., Cai, L., Li, D., Wang, X., Zhao, S., Zou, F., & Zhou, K. (2018). Metagenomics Biomarkers Selected for Prediction of Three Different Diseases in Chinese Population. BioMed Research International, 2018. https://doi.org/10.1155/2018/2936257
48	Chen	The prognostic value of whole-genome DNA methylation in response to Leflunomide in patients with Rheumatoid Arthritis	2023	Predict Treatment Response	Logistic Regression, Random Forest, Boosted Tree, SVM, Naïve Bayes	77	NA	NA	0	0	0	1	0	1	2	1	1	0	0	1	5	Chen, Y., Wang, Q., Liu, H., Jin, L., Feng, X., Dai, B., Chen, M., Xin, F., Wei, T., Bai, B., Fan, Z., Li, J., Yao, Y., Liao, R., Zhang, J., Jin, X., & Fu, L. (2023). The prognostic value of whole-genome DNA methylation in response to Leflunomide in patients with Rheumatoid Arthritis. Frontiers in Immunology, 14. https://doi.org/10.3389/fimmu.2023.1173187
49	Okita	Automatic evaluation of atlantoaxial subluxation in rheumatoid arthritis by a deep learning model	2023	Assess Joint Damage	NN	78	NA	NA	0	0	0	0	0	0	0	1	0	1	1	2	5	Okita, Y., Hirano, T., Wang, B., Nakashima, Y., Minoda, S., Nagahara, H., & Kumanogoh, A. (2023). Automatic evaluation of atlantoaxial subluxation in rheumatoid arthritis by a deep learning model. Arthritis Research and Therapy, 25(1). https://doi.org/10.1186/s13075-023-03172-x
50	Rao	Machine Learning Approaches to Classify Self-Reported Rheumatoid Arthritis Health Scores Using Activity Tracker Data: Longitudinal Observational Study	2023	Score Disease Activity	Random Forest, Other	92	NA	NA	0	0	0	0	0	0	1	0	0	1	1	1	4	Rao, K., Speier, W., Meng, Y., Wang, J., Ramesh, N., Xie, F., Su, Y., Nowell, W. B., Curtis, J. R., & Arnold, C. (2023). Machine Learning Approaches to Classify Self-Reported Rheumatoid Arthritis Health Scores Using Activity Tracker Data: Longitudinal Observational Study. JMIR Formative Research, 7. https://doi.org/10.2196/43107
51	Rothe	Fluorescence optical imaging feature selection with machine learning for differential diagnosis of selected rheumatic diseases	2023	Improve Diagnostic Accuracy	Boosted Tree	76	NA	NA	0	0	0	0	0	0	2	1	0	1	0	1	5	Rothe, F., Berger, J., Welker, P., Fiebelkorn, R., Kupper, S., Kiesel, D., Gedat, E., & Ohrndorf, S. (2023). Fluorescence optical imaging feature selection with machine learning for differential diagnosis of selected rheumatic diseases. Frontiers in Medicine, 10. https://doi.org/10.3389/fmed.2023.1228832
52	Saleh	USE OF SOME BONE-RELATED CYTOKINES AS PREDICTORS FOR RHEUMATOID ARTHRITIS SEVERITY BY NEURAL NETWORK ANALYSIS	2023	Score Disease Activity	NN	57	NA	NA	0	0	1	0	0	1	1	1	0	0	1	0	3	Saleh, R. O., Mahmood, L. A., Mohammed, M. A., Al-Rawi, K. F., & Al-Hakeim, H. K. (2023). USE OF SOME BONE-RELATED CYTOKINES AS PREDICTORS FOR RHEUMATOID ARTHRITIS SEVERITY BY NEURAL NETWORK ANALYSIS. Russian Journal of Infection and Immunity, 13(1), 147–155. https://doi.org/10.15789/2220-7619-UOS-2008
