# Supplementary material for: Sex bias consideration in healthcare machine-learning research: a systematic review in rheumatoid arthritis
Source: BMJ Open. 2025 Mar 13;15(3):e086117. doi: 10.1136/bmjopen-2024-086117 (PMC11906982; doi:10.1136/bmjopen-2024-086117)
Supplement: Supplementary file 2 [file bmjopen-2024-086117.reviewer_comments.pdf]

PEER REVIEW HISTORY

BMJ Open publishes all reviews undertaken for accepted manuscripts. Reviewers are asked to complete a checklist review form and are provided with free text boxes to elaborate on their assessment. These free text comments are reproduced below.

ARTICLE DETAILS

Title (Provisional)

Sex Bias Consideration in Healthcare Machine Learning Research: A Systematic Review in Rheumatoid Arthritis

Authors

Talwar, Anahita; Turner, Shruti; Maw, Claudia; Quayle, Georgina; Watt, Thomas N; Gohil, Sunir; Duckworth, Emma; Ciurtin, C

VERSION 1 - REVIEW

**Reviewer**	**1**
**Name**	**Chen, Hong**
**Affiliation**	**Southeast University Zhongda Hospital**
**Date**	**07-Jun-2024**
**COI**	**None.**

This is a systematic review with novel topics and rigorous methods. However, to further improve the quality of the article, I suggest that the author can also make some additions and improvements in the following aspects:

1. The author chooses rheumatoid arthritis as the research object of gender bias in machine learning, and draws many valuable findings. However, it remains to be seen whether these findings can be generalized to other diseases. It is suggested that the authors further discuss the potential implications of the conclusions of this review for machine learning research in other disease fields, as well as disease-specific factors that need to be paid attention to in conducting similar research. This will help to strengthen the external validity of the results of this study.

2. In the discussion section, the author presents some hypotheses and valuable insights to explain the current situation. On this basis, it is suggested that the author further systematically sort out and look into the specific breakthrough points and key issues of future research.

**Reviewer**	**2**

**Name**	**Petersen, Eike**
**Affiliation**	**Fraunhofer Institute for Digital Medicine MEVIS**
**Date**	**13-Jun-2024**
**COI**	**None**

The authors investigate the treatment of sex bias in healthcare ML, with a focus on rheumatoid arthritis, specifically. This particular disease represents an interesting case study, since RA is subject to clear sex representation bias and there are also important biological differences in male and female RA. The authors perform a systematic survey in this area, finding that many papers do not even provide disaggregated model performance per sex, not to speak about subtler issues such as utilized variables not being equally useful for men and women.

I commend the authors on their choice of topic, careful analysis, and generally very clear writing. RA indeed shines an interesting spotlight on this important issue - sex bias really does not come much more obvious than here. If folks do not even (care to?) notice the bias here, how will they ever catch its subtler instances?

I have one key criticism, and it concerns the different types of bias which the authors consider. Are these taken from somewhere else (no reference is provided) or "made up" by the authors? My issue with the present definitions is twofold.

i) Especially for a formal analysis of the literature such as performed in this manuscript, referring to an established taxonomy of bias types should be preferred instead of making up even more definitions. Such taxonomies abound, see e.g. https://dl.acm.org/doi/abs/10.1145/3457607, https://www.iso.org/standard/77607.html, https://doi.org/10.6028/NIST.SP.1270, https://courses.cs.duke.edu/spring20/compsci342/netid/readings/suresh-guttag-framework.pdf, https://www.nature.com/articles/s42256-024-00797-8.

ii) The definitions employed here seem imprecisely specified to me. What does it mean that input and output variables "may be biased in sex representation"? In Table 2, this suddenly becomes "variables that better represent a particular sex", which is really quite different! From the later sections of the manuscript, I gather that by this the authors simply mean that the distributions of those variables might be different in men / women? But is this even a bias if e.g. disease prevalence differs between men and women? Shouldn't these tests at the very least be conditioned on the disease status? And are these categories also meant to capture cases such as label / measurement bias or the situation in which a variable is simply more informative in one (sex) group vs. another? (See e.g. https://www.nature.com/articles/s41591-020-01192-7, https://www.science.org/doi/10.1126/science.aax2342, https://dl.acm.org/doi/10.1145/3442188.3445901.)

I would urge the authors to use very clear and precise bias definitions, whichever specific definitions they decide to pursue. (Inserting some mathematical formulae into these definitions probably isn't strictly necessary, but it also wouldn't hurt.)

Minor issues follow.

The authors write "A common source of bias arises when certain groups are underrepresented in the

training and test data. Consequently, model predictions will suffer for such underrepresented groups." This is not a safe implication. At least theoretically, it may even be beneficial for a group to be underrepresented; this all depends on how exactly the groups differ. See e.g. https://www.cell.com/patterns/fulltext/S2666-3899(23)00145-9.

In Table 1, it took me a while to figure out that the green rows are the inclusion criteria and the white ones the exclusion. The whole table is very ugly overall.

In Table 3, is it really clear that not using ensembling is *always* bad (and hence should give 0 points)? If that is the case, then why does one have to justify ensembling to go from 1 to 2 points?

For the discussion: This manuscript fits neatly into a by now quite comprehensive body of literature demonstrating widespread shoddy ML research, e.g. https://www.bmj.com/content/369/bmj.m1328, https://doi.org/10.1177/2167702620954216, https://doi.org/10.1016/j.jclinepi.2019.02.004, https://doi.org/10.1016/j.patter.2023.100804. These topics aren't new; (some) solutions and best practices have been known for years by now. Maybe this is beside the point of this piece, but it does beg the question: what, if anything, needs to change?

**Reviewer**	**3**
**Name**	**Arellano-Avendaño, Francisco Javier**
**Affiliation**	**Benemérita Universidad Autónoma de Puebla Facultad de Medicina**
**Date**	**04-Nov-2024**
**COI**	**-----**

Regarding the manuscript titled "Investigating Sex Bias in Machine Learning Research: A Systematic Review in Rheumatoid Arthritis," the following considerations are presented:

The title does not adequately encompass the entirety of the research.

The abstract lacks clear sectioning and contains ambiguous statements in several instances.

The text exhibits errors in syntax as well as grammatical issues.

The study cannot be replicated due to the omission bias arising from the exclusion of certain manuscripts based on technical language, which may interfere with the significance of the results.

The results are not described with sufficient clarity. While the guidelines for identifying errors are evident, there is an emphasis on their absence without analyzing the researchers' errors.

The descriptive statistical analysis is not clearly articulated.

Syntax and grammatical errors were identified; with the appropriate markings, it is suggested that the author improve the quality of the writing.

The reviewer provided a marked copy with additional comments. Please contact the publisher for full details.

VERSION 1 - AUTHOR RESPONSE

Reviewer: 1
Prof. Hong Chen, Southeast University Zhongda Hospital
Comments to the Author:
This is a systematic review with novel topics and rigorous methods. However, to further improve the quality of the article, I suggest that the author can also make some additions and improvements in the following aspects:

1. The author chooses rheumatoid arthritis as the research object of gender bias in machine learning, and draws many valuable findings. However, it remains to be seen whether these findings can be generalized to other diseases. It is suggested that the authors further discuss the potential implications of the conclusions of this review for machine learning research in other disease fields, as well as disease-specific factors that need to be paid attention to in conducting similar research. This will help to strengthen the external validity of the results of this study.

We appreciate your insightful feedback regarding the generalisability of our findings. To address this, we have added the following section to the discussion that explores generalisability to other disease areas, and to other underrepresented or minority groups:

“While this study aimed to shed light on the specific issue of sex bias in the context of RA, a comprehensive assessment of bias in healthcare algorithms demands a broader perspective. For instance, conditions such as cardiovascular disease, diabetes, and cancer also exhibit sex differences in prevalence and outcomes, which could similarly influence the presence and impact of gender bias in ML models. Notably, women are often underrepresented in clinical trials and research datasets, potentially leading to biases in diagnostic and treatment algorithms even in areas without sex-specific differences. Moreover, existing literature suggests biases in ML have negative impacts on other underrepresented or minority groups, including racial and ethnic minorities[81,82]. Increased consideration of the types of bias outlined here are imperative to ensuring the inclusion of diverse and representative data is critical for the development of equitable

and accurate models to enhance the fairness and reliability of ML applications in healthcare."

2. In the discussion section, the author presents some hypotheses and valuable insights to explain the current situation. On this basis, it is suggested that the author further systematically sort out and look into the specific breakthrough points and key issues of future research.

We appreciate your insightful feedback regarding the need to systematically identify and explore specific breakthrough points and key issues for future research. To address this, we have expanded the discussion section to address our key recommendations:

"Addressing the complexity of sex bias in ML models for RA can be effectively managed using known mitigation strategies. These strategies include the use of diverse and representative datasets, reweighting models, and employing advanced tools such as the Health Equity Assessment Toolkit[72] and fairness detector libraries like AIF360[73] or TensorFlow[74]. Techniques such as re-sampling (e.g., SMOTE), adjusting decision thresholds for different sex groups, stratified cross-validation, and creating sex-specific models are well-documented in the literature and have been shown to improve predictive accuracy and fairness across sexes[75,76]. Despite the availability of these mitigation strategies, the primary challenge lies in raising awareness and ensuring their widespread adoption among researchers. To address this issue, the implementation of standardized guidelines is crucial. The "Sex and Gender Equity in Research" guidelines[77] provide a comprehensive framework for reporting sex and gender considerations in research, while the "Guidelines for Accurate and Transparent Health Estimates Reporting"[78] offer valuable recommendations for ensuring transparency and accuracy in health research. These guidelines, endorsed by leading organizations, can serve as essential tools for researchers to systematically address sex bias in their studies."

Reviewer: 2
Dr. Eike Petersen, Fraunhofer Institute for Digital Medicine MEVIS
Comments to the Author:
The authors investigate the treatment of sex bias in healthcare ML, with a focus on rheumatoid arthritis, specifically. This particular disease represents an interesting case study, since RA is subject to clear sex representation bias and there are also important biological differences in male and female RA. The authors perform a systematic survey in this area, finding that many papers do not even provide disaggregated model performance per sex, not to speak about subtler issues such as utilized variables not being equally useful for men and women.

I commend the authors on their choice of topic, careful analysis, and generally very clear writing. RA indeed shines an interesting spotlight on this important issue - sex bias really does not come much more obvious than here. If folks do not even (care to?) notice the bias here, how will they ever catch its subtler instances?

I have one key criticism, and it concerns the different types of bias which the authors

consider. Are these taken from somewhere else (no reference is provided) or "made up" by the authors? My issue with the present definitions is twofold.
i) Especially for a formal analysis of the literature such as performed in this manuscript, referring to an established taxonomy of bias types should be preferred instead of making up even more definitions. Such taxonomies abound, see e.g. https://dl.acm.org/doi/abs/10.1145/3457607, https://www.iso.org/standard/77607.html, https://doi.org/10.6028/NIST.SP.1270, https://courses.cs.duke.edu/spring20/compsci342/netid/readings/suresh-guttag-framework.pdf, https://www.nature.com/articles/s42256-024-00797-8.

Thank you for your valuable feedback. We appreciate the opportunity to clarify our approach to establishing the bias matrix and apologise for any confusion caused by the incomplete explanation.
In our study, we aimed to create a bias matrix that could fulfil three main criteria:

1. **Relevance to Our Specific Research Question**: To achieve this, we selected bias measures from previous literature that were most relevant to our research aims. Specifically, we referenced a framework focused on sex and gender bias in ML healthcare: Nature Article, rather than the more general ML bias frameworks mentioned by the reviewer. However, we note that this framework is very similar to the one in the article referenced by the reviewer: Duke Course Reading.
2. **Ease of Implementation within a Systematic Review Context**: Our research was a systematic review, meaning that our bias matrix had to be consistently applied to a number of diverse research papers. We included further restrictions, such as our decision to focus on supervised machine learning methodologies. To fulfill this aim, we selected the bias measures that we believed could be most uniformly applied in the context of systematic reviews. This adaptation allowed us to provide a more precise and relevant analysis for our specific research focus.
3. **Clarity and Intuitiveness for Our Readers**: To enhance clarity and intuitiveness, we renamed the selected bias measures.

We have now clarified these choices and referenced the aforementioned bias matrices in the methods section, under Data Extraction, keeping the explanation concise due to word count limitations:

"Our bias matrix was adapted from a framework focused on sex and gender bias in ML healthcare[44] and tailored to be relevant and implementable in our systematic review context, and intuitive for readers."

ii) The definitions employed here seem imprecisely specified to me. What does it mean that input and output variables "may be biased in sex representation"? In Table 2, this suddenly becomes "variables that better represent a particular sex", which is really quite different! From the later sections of the manuscript, I gather that by this the authors simply mean that the distributions of those variables might be different in men / women? But is this even a bias if e.g. disease prevalence differs between men and women? Shouldn't these tests at the very least be conditioned on the disease status? And are these categories also meant to capture cases such as label / measurement bias or the situation in which a variable is simply more informative in one (sex) group vs.

another? (See e.g. https://www.nature.com/articles/s41591-020-01192-7, https://www.science.org/doi/10.1126/science.aax2342, https://dl.acm.org/doi/10.1145/3442188.3445901.)

I would urge the authors to use very clear and precise bias definitions, whichever specific definitions they decide to pursue. (Inserting some mathematical formulae into these definitions probably isn't strictly necessary, but it also wouldn't hurt.)

Thank you for your detailed feedback. We appreciate your emphasis on the need for clear and precise definitions of bias. We acknowledge that our initial definitions may have seemed imprecise and could benefit from further clarification and consistency.

When we describe potential bias in input and output variables, we mean that the distributions of these variables might differ between men and women due to biological differences, disease prevalence, and other socio-demographic factors. We agree that it is essential to condition this on disease status to ensure that any observed differences in variable distributions are not merely due to differences in disease prevalence between sexes. Finally, we acknowledge that variable bias can be compounded by label/measurement bias, which occurs when there are systematic errors in the way data is labelled or measured. However, these types of biases can be challenging to separate precisely, especially in the context of a systematic review, assessing papers written by different authors. Therefore, we have grouped them under the broader category of variable biases but highlighted that they may be relevant.

We have revised our definitions to incorporate these nuances, and updated Table 2 with the same definitions for consistency.

Revised Definitions and Clarifications

1) **Training Data Bias**: This bias stems from sex skew in the training datasets, leading to models that may perform differently for men and women.
2) **Test Data Bias**: This bias originates from sex skew in the test dataset. It affects the evaluation of the model's performance, potentially leading to misleading conclusions about its effectiveness across sexes.
3) **Input Variable Bias**: This bias arises from input variables that may have different distributions or levels of informativeness between sexes, conditioned on disease status. It may be compounded by label/measurement bias, which occurs when there are systematic errors in the way data is labelled or measured.
4) **Output Variable Bias**: This bias is associated with output variables that may have different distributions or levels of informativeness between sexes, conditioned on disease status. It may be compounded by label/measurement bias.
5) **Analysis Bias**: This bias results from the omission of sex-disaggregated analyses, potentially masking important variations and leading to biased conclusions.

Minor issues follow.

The authors write "A common source of bias arises when certain groups are underrepresented in the training and test data. Consequently, model predictions will

suffer for such underrepresented groups." This is not a safe implication. At least theoretically, it may even be beneficial for a group to be underrepresented; this all depends on how exactly the groups differ. See e.g. https://www.cell.com/patterns/fulltext/S2666-3899(23)00145-9.

We appreciate your attention to the nuances of bias and representation in training and test data. We acknowledge that the statement "A common source of bias arises when certain groups are underrepresented in the training and test data. Consequently, model predictions will suffer for such underrepresented groups." may not fully capture the complexity of the issue. It is indeed possible that underrepresentation could, in some cases, lead to different outcomes, depending on how the groups differ and how the model is trained.

We have revised the statement as follows:
"A common source of bias arises when certain groups are underrepresented in training and test data, contributing to biased model predictions[18,19]. Whilst the impact of this underrepresentation is complex and context-dependent, influenced also by group differences and model training methods, it may result in poorer performance for these groups[20]"

In Table 1, it took me a while to figure out that the green rows are the inclusion criteria and the white ones the exclusion. The whole table is very ugly overall.

Thank you for your feedback regarding Table 1. We understand that the original format was unclear and visually unappealing. To address your concerns, we have split the inclusion and exclusion criteria into separate columns for each category. This change enhances clarity and makes it easier to distinguish between the two types of criteria. We have also improved the overall visual design of the table to make it more aesthetically pleasing and user-friendly.

Table 1. Inclusion and exclusion criteria for paper screening. The first letter of the code indicates the category of the criteria with T: Type, S: Scope, P: Participants, C: Context. The codes "IN" and "EX" represent inclusion and exclusion criteria, respectively, and the numbers following IN or EX (e.g., 1, 2, 3) uniquely identify each specific criterion within its category.

	**Inclusion**	**Exclusion**
**Type**	**T_IN_1:** Original research studies.	**T_EX_1:** Review papers, meta-analyses, preprints, conference abstracts, inaccessible.
**Scope**	**S_IN_1:** Studies developing supervised machine learning models for the prediction of clinically relevant outcomes in rheumatoid arthritis.	**S_EX_1:** Studies applying existing machine learning models without developing new models in the domain of rheumatoid arthritis.
		**S_EX_2:** Studies lacking substantial focus on supervised machine learning methods including those utilising text

		mining, unsupervised learning, or those reporting competition results.
		**S_EX_3:** Studies that integrate machine learning methods in rheumatoid arthritis without a primary focus on predicting clinically relevant outcomes.
**Participants**	**P_IN_1:** Studies including human participants with a confirmed diagnosis of any form of rheumatoid arthritis, diagnosed using recognised diagnostic criteria.	**P_EX_1:** Studies not involving humans (e.g. animals, in vitro/ex vivo models, pharmacological agents).
		**P_EX_2:** Studies where the primary focus is not on rheumatoid arthritis (e.g. only healthy controls, involves other autoimmune or musculoskeletal condition outcomes, focus on comorbidities).
**Context**		**C_EX_1:** Studies that do not mention sex.
		**C_EX_2:** Studies that include exclusively male or female participants.

In Table 3, is it really clear that not using ensembling is *always* bad (and hence should give 0 points)? If that is the case, then why does one have to justify ensembling to go from 1 to 2 points?

We appreciate your feedback and agree with your assessment that not using ensembling is *not* inherently bad in all cases. Given the valid scenarios where ensembling might not be necessary or practical, we have decided to remove the ensembling criterion from our quality matrix, and adjusted our scoring criteria and results accordingly. This modification did not impact the final results presented in the manuscript. We believe this adjustment will provide a more balanced and context-sensitive evaluation of model quality.

For the discussion: This manuscript fits neatly into a by now quite comprehensive body of literature demonstrating widespread shoddy ML research, e.g. https://www.bmj.com/content/369/bmj.m1328, https://doi.org/10.1177/2167702620954216, https://doi.org/10.1016/j.jclinepi.2019.02.004, https://doi.org/10.1016/j.patter.2023.100804. These topics aren't new; (some) solutions and best practices have been known for years by now. Maybe this is beside the point of this piece, but it does beg the question: what, if anything, needs to change?

We acknowledge the importance of placing these results in the context of the broader literature. We have expanded our discussion section to incorporate a section that comments on the generalisability of our results, and a section that identifies and explores specific breakthrough points and key issues for future research. Please see responses to reviewer 1 for a more thorough description of the changes.

Reviewer: 3
Dr. Francisco Javier Arellano-Avendaño, Benemérita Universidad Autónoma de Puebla Facultad de Medicina
Comments to the Author:
Regarding the manuscript titled "Investigating Sex Bias in Machine Learning Research: A Systematic Review in Rheumatoid Arthritis," the following considerations are presented:

The title does not adequately encompass the entirety of the research.

We understand the importance of having a title that accurately reflects the scope and content of the research and have updated it to "Sex Bias Consideration in Healthcare Machine Learning Research: A Systematic Review in Rheumatoid Arthritis". This title better emphasises the primary focus on consideration of sex bias by researchers in healthcare machine learning research, using rheumatoid arthritis as an example.

The abstract lacks clear sectioning and contains ambiguous statements in several instances.

Thank you for your feedback. We have revised the abstract with clear sectioning and improved clarity:

Background: Unchecked sex bias in machine learning (ML) algorithms used in healthcare can exacerbate disparities in predictions of disease activity, prognosis and treatment when applied in clinical practice.
Objective: To assess the acknowledgment and mitigation of sex bias within studies using supervised ML for improving clinical outcomes in rheumatoid arthritis (RA).
Design: A systematic review of original studies published in English between 2018 and November 2023. This study is registered on PROSPERO with ID CRD42023431754.
Data Sources: PUBMED and EMBASE databases.
Study Selection: Studies were selected based on their use of supervised ML in RA and their publication within the specified date range.
Data Extraction and Synthesis: Papers were scored on whether they reported, attempted to mitigate, or successfully mitigated various types of bias: training data bias, test data bias, input variable bias, output variable bias, and analysis bias. The quality of ML research in all papers was also assessed.
Results: Out of 52 papers included in the review, 51 had a female skew in their study participants. However, 42 papers did not acknowledge any potential sex bias. Only 3 papers assessed bias in model performance by sex disaggregating their results. Potential sex bias in input variables was acknowledged in 1 paper, whilst 6 papers commented on sex bias in their output variables, predominantly disease activity scores. No paper attempted to mitigate any type of sex bias.
Conclusions: The findings demonstrate the need for increased promotion of inclusive and equitable ML practices in healthcare to address unchecked sex bias in ML algorithms.

The text exhibits errors in syntax as well as grammatical issues.

We thank the reviewer for their detailed comments on our writing. These have been addressed in line.

The study cannot be replicated due to the omission bias arising from the exclusion of certain manuscripts based on technical language, which may interfere with the significance of the results.

We acknowledge your concern regarding the potential omission bias arising from the exclusion of certain manuscripts based on technical language.

Our search strategy and scope were defined to identify research studies using supervised machine learning in the domain of rheumatoid arthritis. Whilst we acknowledge that this may have led to the exclusion of certain manuscripts according to techniques used or language describing them, this is a recognised limitation of systematic reviews. This point has been highlighted as a limitation in the discussion section of our manuscript, and thus we feel it has been reasonably addressed.

Despite the potential omission bias, we believe that the findings of our review remain significant. The majority of the included studies did not acknowledge or mitigate sex bias, highlighting a critical gap in current ML research practices in healthcare. This underscores the need for increased awareness and promotion of inclusive and equitable ML practices.

The results are not described with sufficient clarity. While the guidelines for identifying errors are evident, there is an emphasis on their absence without analyzing the researchers' errors.

Thank you for your valuable feedback regarding the clarity of the results section in our manuscript.

Whilst there is a wealth of literature the impact of bias in ML and healthcare, as well as mitigation techniques, our study aimed to fill a gap by specifically evaluating whether and in what ways researchers in the healthcare domain are addressing sex bias in their ML models for RA.

In response to your feedback, we have added to the discussion section to better contextualise our results within the broader literature on bias mitigation and bias impact. This includes placing our findings within the context of existing research on bias mitigation and discussing the implications for clinical practice and future research, as well as identifying gaps in the current practice of addressing sex bias in ML research and providing recommendations for improvement.

"Addressing the complexity of sex bias in ML models for RA can be effectively managed using known mitigation strategies. These strategies include the use of diverse and representative datasets, reweighting models, and employing advanced tools such as the Health Equity Assessment Toolkit[72] and fairness detector libraries like AIF36073 or TensorFlow[74]. Techniques such as re-sampling (e.g., SMOTE), adjusting decision

thresholds for different sex groups, stratified cross-validation, and creating sex-specific models are well-documented in the literature and have been shown to improve predictive accuracy and fairness across sexes[75,76]. Despite the availability of these mitigation strategies, the primary challenge lies in raising awareness and ensuring their widespread adoption among researchers. To address this issue, the implementation of standardized guidelines is crucial. The "Sex and Gender Equity in Research" guidelines[77] provide a comprehensive framework for reporting sex and gender considerations in research, while the "Guidelines for Accurate and Transparent Health Estimates Reporting"[78] offer valuable recommendations for ensuring transparency and accuracy in health research. These guidelines, endorsed by leading organizations, can serve as essential tools for researchers to systematically address sex bias in their studies."

The descriptive statistical analysis is not clearly articulated.
We have clarified the descriptive analysis in the results section, and added the following section to the methods:

"Data Analysis
The Pearson's correlation coefficient and associated p-value between the quality scores and the consideration of sex bias scores was also calculated."

Syntax and grammatical errors were identified; with the appropriate markings, it is suggested that the author improve the quality of the writing.

We have carefully reviewed the manuscript to address the syntax and grammatical errors identified.

Of note, all numbers referring to counts of papers were edited to be in numeric form and removed from the beginning of sentences. For other numbers in the text, we followed standard scientific writing rules such as specified here:
https://www.aje.com/arc/editing-tip-using-numbers-scientific-manuscripts/

With the appropriate markings provided, we have made the necessary corrections to improve the quality of the writing. We believe these revisions enhance the readability and clarity of the manuscript.

VERSION 2 - REVIEW

**Reviewer**	**2**
**Name**	**Petersen, Eike**
**Affiliation**	**Fraunhofer Institute for Digital Medicine MEVIS**
**Date**	**13-Jan-2025**

COI

I would like to thank the authors for their quite thorough revision of this manuscript. In my opinion, this has already greatly improved the quality of the piece.

My concerns from the previous round have been well-addressed. I have a few minor additional remarks.

1) In the quality assessment matrix, there is a "Cross-Validation" category. Cross-Validation (CV) is a specific methodology for accurately assessing model performance on the same dataset on which the model is also trained. There are many studies in which cross-validation does not need to (and even should not) be applied. To get a score 2 here, the table says "used new sample", which is simply not compatible with using CV, so I venture to guess that the category is misnamed and should be called something else. "Validation data source" or something like that?

2) In the same matrix, there is an "Evaluation" category, where studies obtain 0 points if "not done or accuracy metric only" and 1 point if "accuracy metric plus error or calibration". What is meant by "error" here? The total error rate is literally 1-accuracy, so it would seem a bit strange to award 1 point for calculating that separately. If something else is meant (e.g. sensitivity/specificity), this should be specified more clearly.

3) A bit further below, there is a list of ML categories into which approaches are grouped. I was going to comment that it is an error to force papers into exclusive categories including "Regression", "Neural Networks", "Random Forests", "Gaussian Process" etc. since any of the latter modeling approaches can be used to implement both classification and regression. However, I then realized that the numbers add up to more than the number of papers, so I assume that the categories are not treated as exclusive, and the same paper can be assigned multiple categories? This should be clarified. If categories are non-exclusive, I would still find it strange to have a "regression" category but not also a "classification" category, and possibly other task-specific - as opposed to algorithm-specific - categories.

4) Finally, several comment on some of the newly added content in the discussion. The authors now write that "easily implementable data balancing mitigation methods" are available, that "the complexity of sex bias in ML models [...] can be effectively managed using known mitigation strategies" and that "Techniques such as [some mitigation strategies] are well-documented in the literature and have been shown to improve predictive accuracy and fairness across sexes." While they are indeed readily available, I would recommend a more cautious stance towards these bias mitigation methods.

- Several comprehensive studies have found that essentially no in-processing bias mitigation technique consistently outperforms ERM or balanced ERM in terms of resulting model bias, see e.g. https://openreview.net/pdf?id=6ve2CkeQe5S or https://proceedings.mlr.press/v174/zhang22a.html. Notably, mitigation strategies that involve e.g. improving the training dataset, introducing additional normalization steps,

changing the outcome variable or otherwise changing the task setup can achieve much better results. But what can be achieved in terms of fairness improvements at the purely in-processing stage is really quite limited and always comes with important limitations and trade-offs; there is no simple algorithmic fix for bias issues. Also cf. e.g. https://www.nature.com/articles/s41591-024-03113-4 and https://www.nature.com/articles/s43586-024-00371-x. (Yes, these references are all on medical imaging, but the same results hold true for other domains as well.)

- TensorFlow is certainly no "fairness detector library".

- SMOTE is not designed for imbalanced demographic representation (imbalance in x space) but rather imbalance in outcomes (y), which is different. In addition, it is now relatively widely recognized that there is essentially no theoretical basis for using SMOTE, and that it yields no consistent empirical benefit. See e.g. https://www.sciencedirect.com/science/article/pii/S1532046424000844 and https://academic.oup.com/jamia/article/29/9/1525/6605096.

VERSION 2 - AUTHOR RESPONSE

Reviewer: 2
Dr. Eike Petersen, Fraunhofer Institute for Digital Medicine MEVIS
Comments to the Author:
I would like to thank the authors for their quite thorough revision of this manuscript. In my opinion, this has already greatly improved the quality of the piece.

My concerns from the previous round have been well-addressed. I have a few minor additional remarks.
1) In the quality assessment matrix, there is a "Cross-Validation" category. Cross-Validation (CV) is a specific methodology for accurately assessing model performance on the same dataset on which the model is also trained. There are many studies in which cross-validation does not need to (and even should not) be applied. To get a score 2 here, the table says "used new sample", which is simply not compatible with using CV, so I venture to guess that the category is misnamed and should be called something else. "Validation data source" or something like that?

Thank you for your valuable feedback. Given your observation, it appears that the current naming of the category might lead to confusion. The intention behind the category is to evaluate the source of validation data, whether it is a new sample or the same dataset used for training. Therefore, renaming the category to something more descriptive, such as "Validation Data Source," would indeed provide better clarity and align with the intended assessment criteria.

2) In the same matrix, there is an "Evaluation" category, where studies obtain 0 points if "not done or accuracy metric only" and 1 point if "accuracy metric plus error or calibration". What is meant by "error" here? The total error rate is literally 1-accuracy, so

it would seem a bit strange to award 1 point for calculating that separately. If something else is meant (e.g. sensitivity/specificity), this should be specified more clearly.

Thank you for your insightful comment regarding the "Evaluation" category in the quality assessment matrix. We agree that the term "error" needs to be clearly defined to avoid confusion. In this context, "error" refers to the measurement of uncertainty or variability around the accuracy metric itself. This includes metrics such as confidence intervals (a range of values that is likely to contain the true accuracy of the model, providing an indication of the precision of the accuracy estimate), or standard error (standard deviation of the sampling distribution of the accuracy metric, indicating how much the accuracy estimate would vary if the model were evaluated on different samples).
To address your concern, we have updated the scoring criteria to explicitly mention these additional metrics.

0 Points: Evaluation not done or only accuracy reported.
1 Point: Accuracy plus error metrics of the accuracy (e.g., confidence intervals, standard error) or calibration metrics.
2 Points: Includes accuracy, error metrics of the accuracy, and calibration metrics.

3) A bit further below, there is a list of ML categories into which approaches are grouped. I was going to comment that it is an error to force papers into exclusive categories including "Regression", "Neural Networks", "Random Forests", "Gaussian Process" etc. since any of the latter modeling approaches can be used to implement both classification and regression. However, I then realized that the numbers add up to more than the number of papers, so I assume that the categories are not treated as exclusive, and the same paper can be assigned multiple categories? This should be clarified. If categories are non-exclusive, I would still find it strange to have a "regression" category but not also a "classification" category, and possibly other task-specific - as opposed to algorithm-specific - categories.

Thank you for highlighting this inconsistency. We have amended the Data Synthesis section under Methods to clarify that ML categories are not exclusive.

“The ML algorithms used for clinical prediction in each paper were also extracted and non-exclusively classified into broad categories.”

Further, we have divided the regression category into 2 new categories: linear regression and logistic regression such that all categories are now algorithm-specific, rather than task-specific. The text, supplementary data, and Figure 2 have all been updated accordingly.

4) Finally, several comment on some of the newly added content in the discussion. The authors now write that "easily implementable data balancing mitigation methods" are available, that "the complexity of sex bias in ML models [...] can be effectively managed using known mitigation strategies" and that "Techniques such as [some mitigation strategies] are well-documented in the literature and have been shown to improve predictive accuracy and fairness across sexes." While they are indeed readily available, I

would recommend a more cautious stance towards these bias mitigation methods.
- Several comprehensive studies have found that essentially no in-processing bias mitigation technique consistently outperforms ERM or balanced ERM in terms of resulting model bias, see e.g. https://openreview.net/pdf?id=6ve2CkeQe5S or https://proceedings.mlr.press/v174/zhang22a.html. Notably, mitigation strategies that involve e.g. improving the training dataset, introducing additional normalization steps, changing the outcome variable or otherwise changing the task setup can achieve much better results. But what can be achieved in terms of fairness improvements at the purely in-processing stage is really quite limited and always comes with important limitations and trade-offs; there is no simple algorithmic fix for bias issues. Also cf. e.g. https://www.nature.com/articles/s41591-024-03113-4 and https://www.nature.com/articles/s43586-024-00371-x. (Yes, these references are all on medical imaging, but the same results hold true for other domains as well.)
- TensorFlow is certainly no "fairness detector library".
- SMOTE is not designed for imbalanced demographic representation (imbalance in x space) but rather imbalance in outcomes (y), which is different. In addition, it is now relatively widely recognized that there is essentially no theoretical basis for using SMOTE, and that it yields no consistent empirical benefit. See e.g. https://www.sciencedirect.com/science/article/pii/S1532046424000844 and https://academic.oup.com/jamia/article/29/9/1525/6605096.

Thank you for your feedback. We have adjusted the discussion section to more accurately highlight the nuances you have picked up here:

“Mitigating bias in ML models has been well researched in the literature, with several strategies including the use of diverse and representative datasets, reweighting models, and employing advanced tools such as the Health Equity Assessment Toolkit[72] and fairness detector libraries like AIF360[73]. Techniques such as re-sampling, adjusting decision thresholds for different sex groups, stratified cross-validation, and creating sex-specific models are well-documented in the literature and readily available[74]. While some techniques are available and effective, using balanced data or incorporating representative variables is often the most effective approach for simultaneously maintaining model performance, and as such has been the focus of our review[75–77].”

VERSION 3 - REVIEW

**Reviewer**	**2**
**Name**	**Petersen, Eike**
**Affiliation**	**Fraunhofer Institute for Digital Medicine MEVIS**
**Date**	**18-Feb-2025**
**COI**	

I would like to thank the authors for addressing all of my concerns! I have no further remarks, except that I would like to (again) commend the authors on a very interesting study!
